# Genome-wide distribution of histone trimethylation reveals a global impact of bisphenol A on telomeric binding proteins and histone acetyltransferase factors: a pilot study with human and in vitro data

**DOI:** 10.1186/s13148-022-01408-2

**Published:** 2022-12-26

**Authors:** Shereen Cynthia D’Cruz, Chunxiang Hao, Martin Labussiere, Vicente Mustieles, Carmen Freire, Louis Legoff, Laura Magnaghi-Jaulin, Alicia Olivas-Martinez, Andrea Rodriguez-Carrillo, Christian Jaulin, Arthur David, Mariana F. Fernandez, Fatima Smagulova

**Affiliations:** 1 EHESP, Inserm, Irset (Institut de Recherche en Santé, Environnement et Travail) - UMR_S 1085, University Rennes, 35000 Rennes, France; 2 School of Medicine, Linyi University, Linyi, 276000 China; 3 Center for Biomedical Research (CIBM), Department of Radiology and Physical Medicine, School of Medicine, University of Granada, 18016 Granada, Spain; 4 Instituto de Investigación Biosanitaria (Ibs.GRANADA), 18012 Granada, Spain; 5 Consortium for Biomedical Research in Epidemiology and Public Health (CIBERESP), 28029 Madrid, Spain

**Keywords:** Human blood, DNA methylation, H3K4me3, Telomere, ChIP-seq, Bisphenol A

## Abstract

**Objective:**

To assess the genetic and epigenetic effects promoted by Bisphenol A (BPA) exposure in adolescent males from the Spanish INMA-Granada birth cohort, and in human cells.

**Methods:**

DNA methylation was analysed using MEDIP. Repeat number variation in genomic DNA was evaluated, along with the analysis of H3K4me3 by using chromatin immunoprecipitation followed by high-throughput sequencing (ChIP-seq). Analyses were performed with material extracted from whole blood of the adolescents, complemented by in vitro assessments of human (HeLa) cells exposed to 10 nM BPA, specifically, immunofluorescence evaluation of protein levels, gene expression analysis and ChIP‒qPCR analysis.

**Results:**

Adolescents in the high urinary BPA levels group presented a higher level of Satellite A (SATA) repetitive region copy numbers compared to those in the low BPA group and a tendency towards increase in telomere length. We also observed decreased DNA methylation at the promoters of the imprinted genes *H19, KCNQ1,* and *IGF2*; at *LINE1* retroelements; and at the *ARID2, EGFR* and *ESRRA* and *TERT* genes. Genome-wide sequencing revealed increased H3K4me3 occupancy at the promoters of genes encoding histone acetyltransferases, telomeric DNA binding factors and DNA repair genes. Results were supported in HeLa cells exposed to 10 nM BPA in vitro. In accordance with the data obtained in blood samples, we observed higher H3K4me3 occupancy and lower DNA methylation at some specific targets in HeLa cells. In exposed cells, changes in the expression of genes encoding DNA repair factors (*ATM, ARID2, TRP53*) were observed, and increased expression of several genes encoding telomeric DNA binding factors (*SMG7, TERT, TEN1, UPF1, ZBTB48*) were also found. Furthermore, an increase in ESR1/ERa was observed in the nuclei of HeLa cells along with increased binding of ESR1 to *KAT5, KMT2E* and *TERF2IP* promoters and decreased ESR1 binding at the *RARA* promoter. The DNA damage marker p53/TP53 was also increased.

**Conclusion:**

In this pilot study, genome-wide analysis of histone trimethylation in adolescent males exposed to BPA revealed a global impact on the expression of genes encoding telomeric binding proteins and histone acetyltransferase factors with similar results in HeLa cells. Nevertheless, larger studies should confirm our findings.

**Supplementary Information:**

The online version contains supplementary material available at 10.1186/s13148-022-01408-2.

## Introduction

The number of studies showing negative effects of BPA on human health has dramatically increased in the last decade. As an illustration, a PubMed search with the terms “BPA” and “human” yields more than 5000 results. BPA is a compound used as a monomer in the fabrication of polycarbonate plastics and in epoxy resins to coat the inner lining of cans and beverages among many other applications [1]. BPA is incorporated into the human body mainly through its presence in food packaging products [2], but alternatives routes exist including dust ingestion/inhalation, thermal receipts handling or even through clothing [3, 4]. The main effects of BPA are thought to be attributable to its oestrogenic capacity [5]. Although this effect is very weak, the continued presence of BPA-containing materials in the environment could cause long-lasting adverse health effects [6, 7], which raises serious public concern for public health and especially the health of young people.

In addition to its oestrogenic capacity, BPA can exhibit genotoxic effects. For example, exposure to BPA during mitosis increases sperm chromatin fragmentation and promotes high levels of sperm DNA damage [8]. A high proportion of micronuclei (MN) containing kinetochores has also been detected among cells exposed to BPA. The authors of that study suggested that oestrogens induce MN through interference with the kinase signalling that controls the spindle checkpoint [9]. In addition, it has been shown that the amount of copy number variations (CNVs) in chromosomes 3, 4, 11, 22, and X was higher in BPA-treated neuronal progenitor cells (NPCs) than in control cells. It was suggested that BPA exposure could cause genomic instability in cultured NPCs through the reduction of DNA methyltransferase activity, leading to increased genetic instability [10]. The genotoxic effects of BPA may also be due to oxidative stress through the production of reactive oxygen species (ROS). Indeed, it has been shown that the intraperitoneal injection of BPA induces overproduction of hydrogen peroxide in mouse organs [11], and a significant increase in the DNA damage marker 8-oxoguanosine in urine, after administration of BPA to Sprague–Dawley rats was also observed [12]. Thus, exposure to BPA could lead to oxidative stress, which in turn could lead to genotoxic stress and may cause genome alterations.

Unstable regions are common in mammalian genomes. Classical examples of such unstable regions are satellite sequences. The overexpression of centromeric and pericentromeric satellite repeats has been detected in several cancers [13, 14]. It has been suggested that this unprogrammed satellite DNA expression is mediated via epigenetic deregulations that occur in response to environmental changes or during cell transformation [13]. Yet other unstable regions of the genome were reported, these are the mobile retroelements (REs), which like long interspersed nuclear element 1 (LINE1) are able to integrate into the genome. LINE1 mobility is largely repressed in somatic tissues, but is de-repressed in many tumours [15, 16]. The study showed that LINE1 expression creates specific molecular vulnerabilities and induces a retrotransposition-replication conflict that could contribute to cancer growth [17].

BPA exposure can also induce epigenetic alterations. Thus, prenatal BPA exposure has been shown to induce long-lasting changes in the DNA methylation of the transcriptionally relevant region of the *Bdnf* gene in both the hippocampus and blood of exposed mice. These changes are consistent with *BDNF* DNA methylation changes observed in umbilical cord blood of human newborns exposed to high levels of maternal BPA in utero [18]. Notably, maternal BPA exposure shifted the coat colour distribution of viable yellow agouti (Avy) mouse offspring towards yellow by decreasing CpG methylation at an intracisternal A particle retrotransposon upstream of the Agouti gene [19]. Exposure to BPA could also cause a decrease in DNA methylation at repeated elements as well. For example, a significantly lower level of LINE1 methylation was observed in the sperm of BPA exposed workers (*p* < 0.001) compared to that of non-exposed workers [20]. Moreover, BPA exposure during early stages of embryonic development disrupts imprinted gene expression in embryos and placentas [21]. It is suggested that the main reason that DNA methylation might change would be the activity of the major methyltransferase DNMT1 [22, 23].

BPA exposure can also affect histone marks. Treatment of MCF-7 cells with BPA led to an increase in *EZH2* mRNA expression as well as an increase in EZH2 protein expression [24], suggesting a possible impact on histone H3K27 trimethylation. Furthermore, BPA exposure enhanced age-related spatial cognitive impairment and decreased H3K9ac and H4K8ac in exposed mice [25]. Similarly, another study showed that low-dose BPA exposure caused decreased histone acetylation of H3K9, H3K27 and H4K12 in exposed rats compared to controls [26].

The present study was developed under the framework of the Human Biomonitoring for Europe Initiative (HBM4EU). In this study, we evaluated the possible impacts of BPA exposure in adolescent males from the INMA-Granada cohort (Spain) with the goal of identifying new biomarkers that could be useful in biomonitoring and epidemiological studies. Our hypothesis is that alterations in epigenetic regulation could lead to abnormal gene activity in human subjects exposed to environmentally relevant doses of BPA. Our results show that higher BPA levels are associated with changes in satellite DNA repeat numbers and decreased methylation at imprinted genes, LINE1 retroelements and genes relevant to hormone pathways. Higher BPA levels are also associated with increased H3K4me3 occupancy at the promoters of genes encoding histone acetyltransferases, telomeric DNA binding proteins and DNA repair factors. The results of the epidemiological study were confirmed by in vitro BPA exposure experiments in the human HeLa cell line. Based on our study, a new model of BPA action is proposed.

## Results

### Repeat number variation in adolescent males

**Fig. 1:**
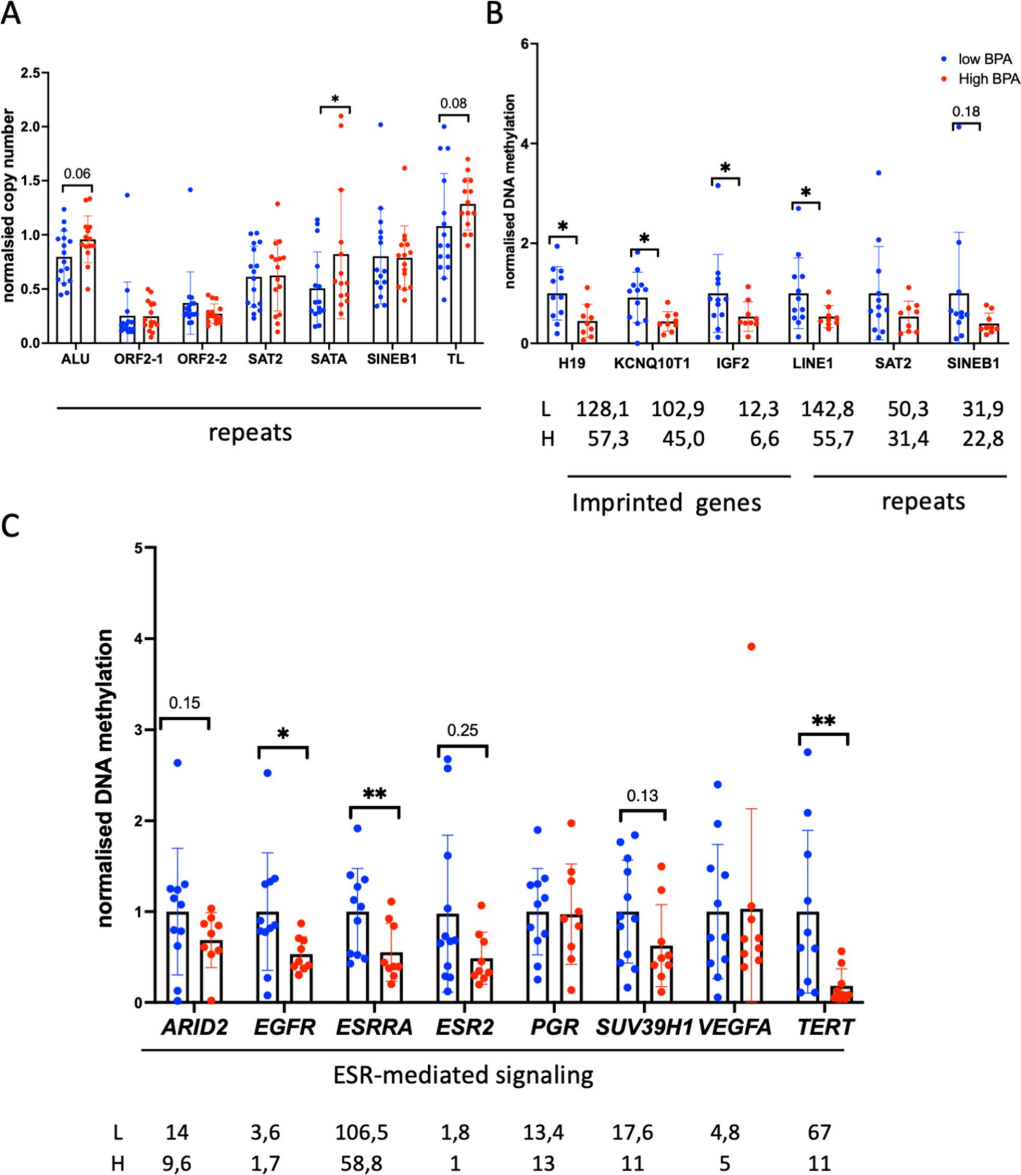
DNA analysis in the blood of adolescent males: **A** Repeat number variations identified in the low BPA and high BPA groups. **B** DNA methylation analysis of imprinted genes and repeat regions. **C** DNA methylation analysis of genes regulated by ESR1. *p < 0.05, Mann‒Whitney test

The experimental design is provided in the Methods section as well as in Additional file 1: Fig. S1. To assess the effects of BPA on genetic stability, we first analysed repeat numbers at variable regions of the human genome: specifically, we looked at the relative repeat numbers of three satellite DNA regions (pericentromeric SAT2, centromeric and pericentromeric SATA), at LINE1 retroelements, which encode for the viral protein ORF2, and at Alu elements, as Alu is the most abundant transposable element. Two groups of LINEs were analysed. The first group (ORF2-1 region) matches 4560 site in the human genome, while the second group (ORF2-2) matches 2918 regions in the genome [27]. We also assessed the telomere repeats. Genomic DNA was extracted from human blood samples, and each DNA sample was adjusted to 0.2 ng/µL. Given the worldwide distribution of BPA, virtually every human has been exposed to some extent. As a consequence, it is not possible to measure the genetic and epigenetic “baseline” in an unexposed control group. Therefore, our analysis was performed by comparing groups with low and high BPA levels. The samples from each group are presented in Additional file 1: Table S1. Repeat numbers were normalized to the *RPLP0* gene. Normalized DNA copy number variations were plotted and compared (Fig. 1). We observed a significant increase in the repeat number of centromeric SATA (fold change (FC) = 1.6, *p* = 0.05) and a tendency to increase for Alu repeat number (FC = 1.2, *p* = 0.06) in the high BPA group (Fig. 1A) (*n* = 15, BPA range 4.85–42.64 µg/g) compared to the low BPA group (*n* = 16, BPA range 0.66–4.67 µg/g, (Additional file 1: Table S1)). We also observed a tendency to an increased telomere length (TL) in the high BPA group (Fig. 1A).

### Analysis of DNA methylation in adolescent males

Next, differences in DNA methylation in the CpG regions of various promoters were analysed in 12 samples from the low BPA group and 9 from the high BPA group. We chose the available samples with the most extreme BPA values for the analysis to reveal the most profound effects of BPA, in low group, *n* = 12, BPA range: 0.66–3.94 µg/g, on high group, *n* = 9, BPA range: 9.44–42.64 µg/g, (Additional file 1: Table S2).

The MEDIP technique was used, and several promoter gene targets were selected for the analysis. To design the primers, the CpG island map of the reference *hg38* genome was downloaded from UCSC using the Table Browser tool, and the Primer-blast tool from PubMed was used. Several control regions were chosen for the MEDIP assay, and a set of imprinted genes (*H19, KCNQ1OT1, IGF2*) was also analysed, as the imprinting of these genes requires DNA methylation. Satellite DNAs (SAT2) and retroelements such as SINEB1 and the 5′UTR of LINE1 were also chosen due to the high levels of DNA methylation in these regions. The ratio of precipitated methylated DNA to input DNA was calculated. Most targets showed strong MEDIP to input ratios, indicating that these targets are highly methylated. The data are shown in Fig. 1B, C. Adolescents in the high BPA group showed lower DNA methylation than those in the low BPA group for all sequences tested, and these differences were statistically significant—approximately 0.5-fold—for the *H19, KCNQ10T1, IGF2* and *LINE1* promoters.

On the other hand, since BPA exhibits oestrogenic activity, we wondered whether DNA methylation would be altered at the promoters of genes that have indications of regulation by ESR1 and have ESR1 binding sites in the vicinity. For this purpose, genes regulated by ESR1 were chosen, and we used the Chea 2022 database of ESR1 binding ChIP-seq data from Enrichr [28]. Thus, we chose to analyse DNA methylation at the promoters of the *ARID2, ESRRA, EGFR, PGR, ESR2, SUV39H1, VEGFA* and *TERT* genes. The results showed a significant decrease in the DNA methylation of *ESRRA* (FC = 0.6, *p* = 0.0096) and *EGFR* (FC = 0.5, *p* = 0.038) and a decreasing trend in *ARID2* (FC = 0.7, *p* = 0.15) and *SUV39H1* (FC = 0.63, *p* = 0.13) in the high BPA group compared to the low BPA group (Fig. 1C). We also analysed DNA methylation at the telomerase (*TERT*), since it is known that oestrogenic compounds affect telomere length (TL); which was corroborated by our results showing a significant decrease of *TERT* DNA methylation in the high BPA group (FC = 0.16, *p* = 0.003), Fig. 1C.

Overall, the above results indicate that high BPA exposure generally correlates with lower DNA methylation. This decrease in DNA methylation was observed at the promoters of genes that regulated by ESR1/ERa, imprinted genes and LINE1 sequences*.*

### Genome-wide analysis of H3K4me3

To investigate the potential new role of BPA in the regulation of the epigenetic landscape at the genome level, genome-wide analysis of H3K4me3 occupancy was performed using 100 µL of whole blood samples from 10 adolescents in the low BPA group, BPA range: 0.66–2.55 µg/g, and 7 adolescents in the high BPA group, BPA range: 10.07–42.64 µg/g (Additional file 1: Table S3). Chromatin immunoprecipitation was performed using anti-H3K4me3 antibody followed by high-throughput sequencing (all details are provided in the Methods section). This histone mark was chosen because it is associated with transcription start sites and its presence correlates well with gene expression. Sequencing reads were analysed, and the differential peaks were detected. Using cut-off values of FC >  = 1.5 and FDR < 0.05, 5707 peaks were found to be differential between the two exposure groups (Additional file 1). The vast majority of differential peaks that we found had increased H3K4me3 occupancy in the high BPA group compared to the low BPA group.

**Fig. 2:**
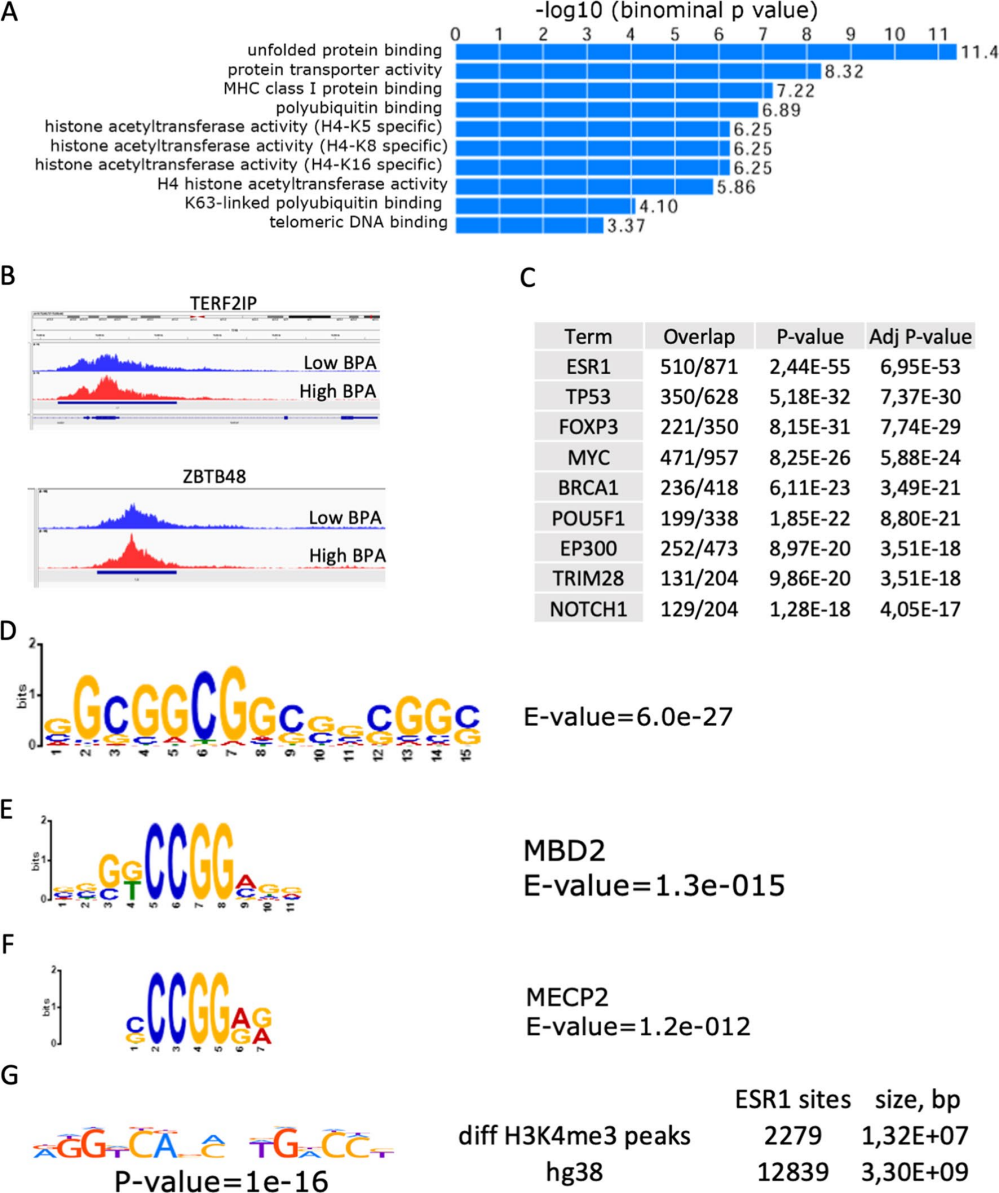
Differential H3K4me3 occupancy in the low and high BPA groups. Functional annotation analysis of differential regions. **A** Gene ontology annotation with “molecular function” terms was performed by GREAT. Terms were sorted by *p* value. **B** H3K4me3 regions in *TERF2IP* and *ZBTB48*. Reads were mapped and plotted on a graph by IGV; blue represents the low BPA group, and red represents the high BPA group. **C** Comparison of genes located in differential peaks and protein‒protein interaction (PPI) analysis; the second column shows the number of genes identified for each transcription factor compared to the total number of known interacting proteins. **D** Motif found by MEME. Parts of this motif are similar to the binding sites of **E** the methyl binding protein MBD2 and **F** the methyl binding protein MECP2. The *E*-value shows the statistical significance of the motif. **G** ESR1 binding sites. The *E*-value shows the statistical significance of the motif presence in differential peaks

Differential H3K4me3 occupancy regions were then screened for genes with shared biological functions. To this end, differential peaks were assigned to genes using GREAT (details and parameters of these analyses are given in the Methods section). A total of 666 genes related to different “biological process” (Additional file 1: Fig. S2A), “molecular function” (Fig. 2A), or “cellular component” (Additional file 1: Fig. S2B) terms were assigned. Interestingly, the “molecular function” analysis revealed H3K4me3 differential occupancy in gene clusters related to histone protein transporter activity, histone H4 acetyltransferase activity, and telomeric DNA binding (Fig. 2A). For example, the enriched cluster ‘H4 histone acetyltransferase activity’ includes genes such as *KAT2A, KAT8,* and *OG,* and the enriched cluster ‘telomeric DNA binding’ contains 22 genes (Additional file 1: Table S4) important for telomere function, such as *PURA, RPA1, TEN1, TERT, TERF2IP* and *ZBTB48.* An example of differential peaks for *TERF2IP* and *ZBTB48* is illustrated in Fig. 2B.

The list of genes identified in differential regions assigned by GREAT was then compared with the Enrichr protein‒protein interaction (PPI) database [28]. This database combines the data of protein‒protein interactions previously described in experimental studies.

This analysis revealed that targets of 9 proteins, including ESR1/ERa, DNA repair factors (BRCA1, TP53), transcriptional factors (MYC, FOXP3, POU5F1, NOTCH1) and chromatin regulators (EP300, TRIM28), were all enriched in our dataset.

Our analysis revealed that genes encoding ESR1-interacting proteins were strongly enriched for H3K4me3 in the high BPA group (Fig. 2C). According to the Enrichr PPI database, ESR1 could interact with 871 proteins, of which 510 were encoded by genes located within differential H3K4me3 peaks (adj. *p* value = 6.95E−53). Among these genes were the *AHR, ESRRA, NCOR1, NCOR2, RARA* and *RXRA* receptors (Additional file 1: Table S5). Furthermore, a PPI database search showed that 236 genes were located in differential peaks corresponding to genes encoding proteins that interact with BRCA1 (BRCA1 could interact with up to 418 proteins). Many BRCA1-interacting proteins encoded by genes located in differential peaks were DNA repair proteins. The AMIGO database includes 519 “DNA repair” genes. Of them, 39 genes were detected in our differential peaks, including *ATM, ATR, BLM, FANCA, MDC1, MLH1, MSH2, MSH3* and *RAD51* (Additional file 1: Table S6). These genes encode proteins critical for various DNA repair processes, suggesting that exposure to BPA could activate DNA repair pathways.

Overall, whole-genome H3K4me3 occupancy analysis revealed that the methylation at genes involved in protecting genetic stability, such as DNA repair genes and telomeric DNA binding protein-encoding genes, was altered by BPA exposure, suggesting the potential toxicity of BPA in these crucial processes.

### Motif analysis in differential H3K4me3 peaks

To address whether regulatory DNA motifs were present within the regions containing the altered peaks, MEME-CHIP was used [29]. We investigated whether there was enrichment for transcription factor-binding sites in the differential peaks. For this, sequences located in differential peaks were extracted in *fasta* format, and repeated masking was performed to remove repetitive sequences from the analysis. The resulting data were processed in MEME-CHIP to reveal the significantly enriched motifs in the differential peaks. A recurrent 15-mer motif was detected by MEME-ChIP (Fig. 2D). Parts of this motif were similar to the MBD2 binding site (*E*-value = 1.3−e15) (Fig. 2E) and the MECP2 binding sites (Fig. 2F).

We also looked for the presence of ESR1 binding sites in the differential H3K4me3 peaks. We used the FIMO tool for ESR1 motif scanning. The ESR1 binding motif was downloaded from HOCOMO (https://hocomoco11.autosome.org). FIMO detected 12,839 sites in the human reference genome *hg38* (*p* value less than 1e−05). Next, we scanned the sequences from differential H3K4me3 peaks for the presence of potential ESR1 binding sites. There were 2279 occurrences of ESR1 motifs with a *p* value less than 1e−05. A comparison of the ESR1 sites found in differential peaks to a total ESR1 sites in the *hg38* reference genome showed a significant increase in ESR1 binding sites in the high BPA group (*p* value = 1e−16, Chi-square test, Fig. 2G) compared to the low BPA group, suggesting a significant impact of BPA exposure on the epigenetic regulation of ESR1 targets.

### Comparison of alterations in histone marks in human samples with gene expression changes in the HeLa cell line

To confirm the observed changes and further mechanistically assess the impacts of BPA on human cells, several in vitro assays were performed in HeLa cells exposed to a low dose of BPA (10 nM), a dose that has not revealed general toxicity [30, 31] and coincides with urinary BPA concentrations showed by the general population (around 2 ng/mL [32]).

**Fig. 3:**
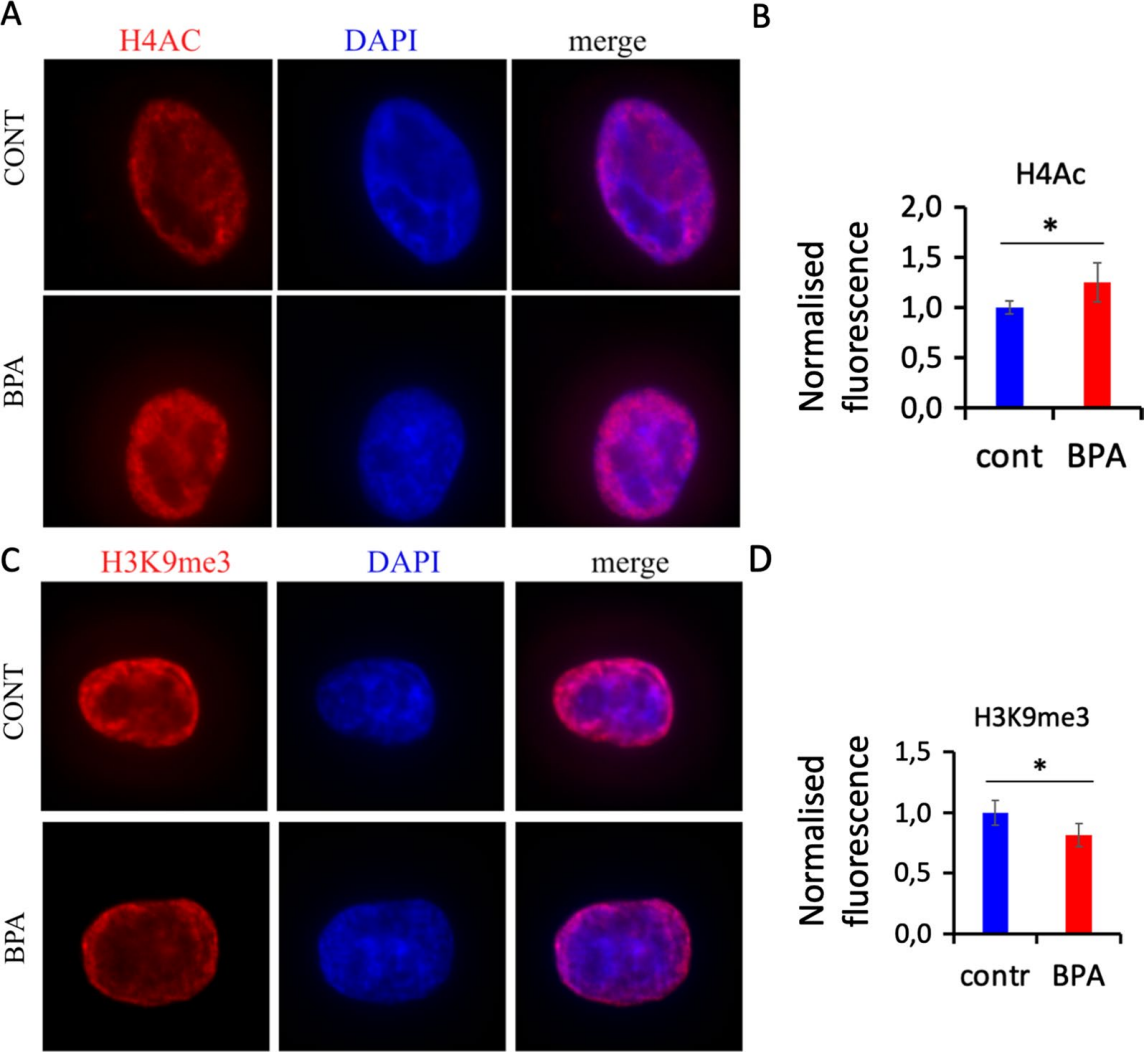
Global histone modification analysis in HeLa cells. **A** H4 acetylation in control cells (top) and in BPA-exposed cells (bottom); **B** Quantitative analysis of H4 acetylation; **C** H3K9me3 in control cells (top) and in BPA-exposed cells (bottom); **D** Quantitative analysis of H3K9me3. The sum of fluorescence for all slices of z-stacks was calculated for each channel. The histone modification signal was normalized to DAPI and presented as combined corrected fluorescence compared to the control, *p < 0.05, Mann‒Whitney test, *n* = 4 experiments for each group

The important observed effects of BPA exposure were on histone H4 acetylation enzymes, so we chose to analyse the levels of histone H4 acetylation which is abundant at open chromatin. We also looked at H3K9 trimethylation, which is enriched at heterochromatin regions. For this analysis, cells were fixed, permeabilized and immunostained against the selected histone marks. Signals were analysed by fluorescence microscopy. To quantitatively analyse the histone mark distribution, z-stack analysis was performed. The z-stack signals were merged and normalized to the merged DAPI signals. We observed that H4 acetylation appeared everywhere throughout the nucleus (Fig. 3A). Quantitative analysis showed a slight but significant 1.2-fold increase in H4 intensity in BPA-exposed cells (Fig. 3B). H3K9me3 was preferentially localized in the cell periphery (Fig. 3C), and in contrast to H4 acetylation, H3K9me3 global amount appeared to be decreased in BPA-exposed cell samples (Fig. 3D).

**Fig. 4:**
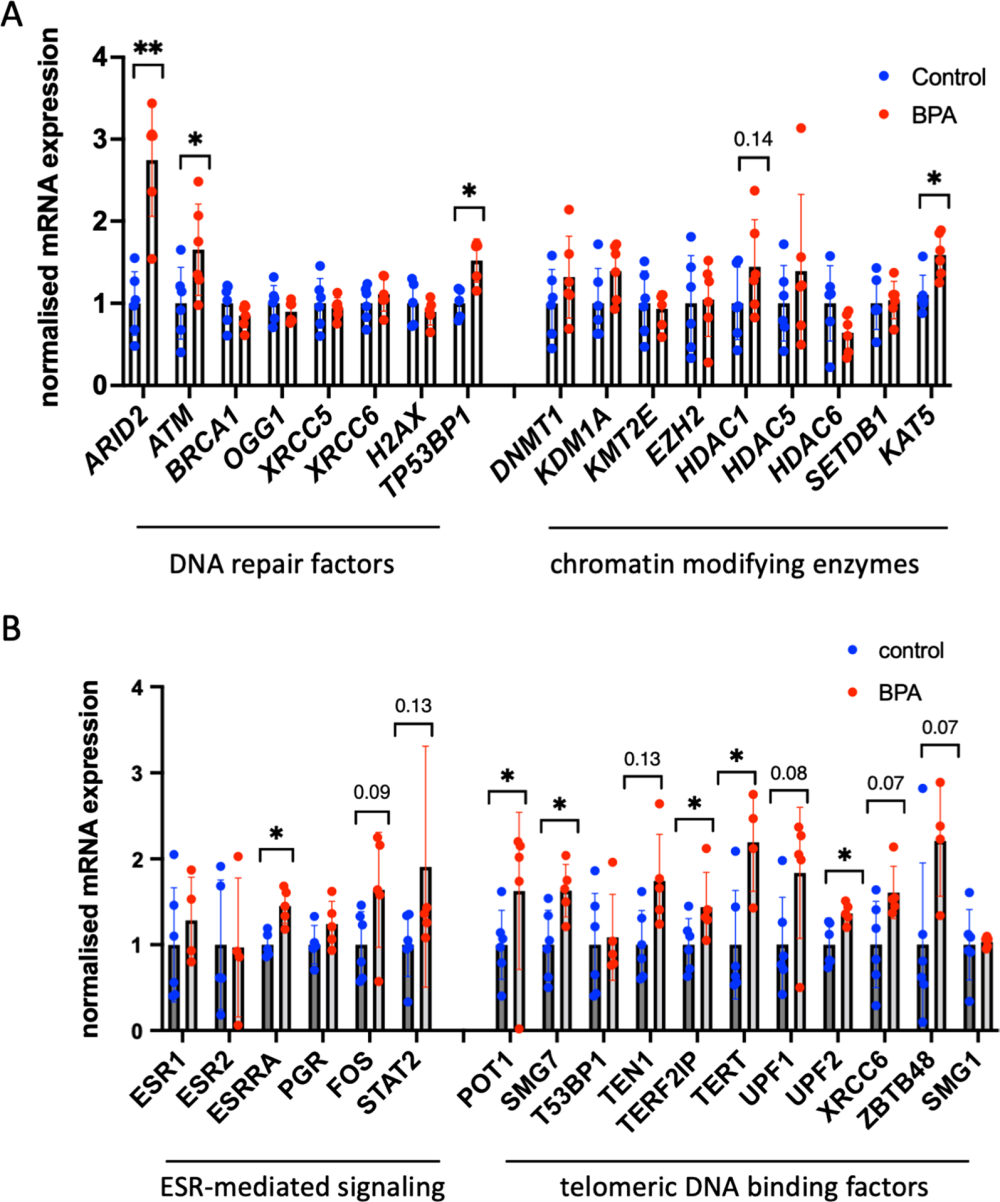
Gene expression analysis by RT‒qPCR in BPA-exposed HeLa cells. **A** Gene expression analysis of DNA repair and chromatin factors. **B** Genes encoding transcription, signalling and telomeric DNA binding proteins, *p < 0.05, **p < 0.01, Mann‒Whitney test, *n* = 6 experiments for each group

Next, we evaluated whether the changes observed in histone H3K4me3 occupancy in human blood samples could lead to gene expression changes in cultured cells. To this end, the expression of genes encoding DNA repair, cell cycle, chromatin remodelling and transcription factors was measured in HeLa cells exposed or not exposed to BPA. The analysis showed 1.7- 2.7–1.5-fold increases in the expression of the *ATM*, *ARID2, TP53BP1* DNA repair genes, respectively, in BPA-exposed compared to nonexposed cells (Fig. 4A). Strikingly, gene expression was found to be increased in almost all of the telomeric-DNA binding factors tested, including 1.5-, 2.1-, 1.7-, 1.3-, 1.7- and twofold increases in the *SMG7, TERT, UPF1, UPF2, TEN1* and *ZBTB48* genes, respectively (Fig. 4B). In addition, we observed a 1.7-fold increase in *STAT2* expression*. STAT2* acts as a transcriptional activator in inflammatory processes [33]. We did not observe changes in genes encoding oestrogen receptors, except for *ESRRA*, which increased in 1.5 times.

Therefore, these data confirmed that BPA exposure of human cell lines leads to alteration of histone marks and changes in gene expression programs. Modifications of the expression of DNA repair, inflammation and telomeric-DNA binding proteins encoding genes correlate with the differences in DNA methylation and H3K4 occupancy previously observed in human blood samples.

### H3K4me3 and H3K9me3 occupancies and DNA methylation state at selected promoters in HeLa cells

**Fig. 5:**
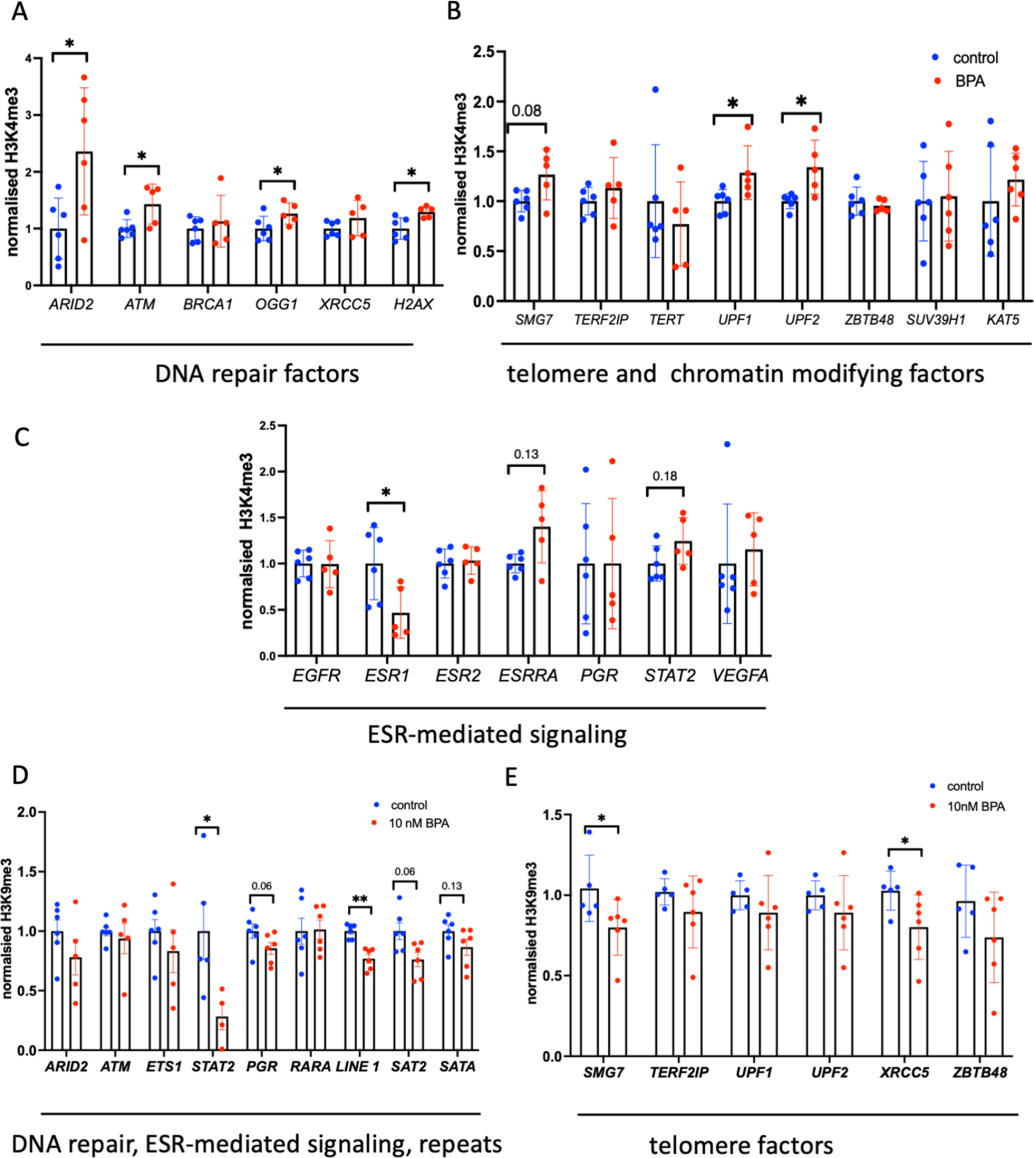
H3K4me3 and H3K9me3 promoter occupancies analysis in HeLa cells. **A** H3K4me3 occupancy at the promoters of DNA repair genes. **B** H3K4me3 occupancy at the promoters of oestrogen signalling genes. **C** H3K4me3 occupancy at the promoters of the telomere maintenance and chromatin factors encoding genes. **D** H3K9me3 occupancy at the promoters of DNA repair and transcription factors genes and at repeated sequences. **E** H3K9me3 occupancy at the promoters of telomere maintenance genes

Next, we asked whether the changes in gene expression were a consequence of changes in epigenetic regulation. We chose to analyse the H3K4me3 marker, as we had analysed it in human samples. We performed chromatin immunoprecipitation against H3K4me3 in exposed and unexposed cells. qPCR analysis was then performed. The qPCR analysis of individual genes showed a global tendency toward increased H3K4me3 occupancy after BPA treatment. The observed increase in H3K4me3 levels was especially significant for the DNA repair genes, including 2.4-, 1.4-, 1.3- and 1.3-fold increases in the promoters of *ARID2, ATM, OGG1* and *H2AX,* respectively (Fig. 5A); and also, for the telomeric-DNA binding factors, including 1.3- and 1.3-fold increases in the promoters of *UPF1* and *UPF2* genes, respectively. Finally, we determined 1.2-fold increase in H3K4me3 at the promoter of *KAT5* (Fig. 5B) and 0.5-fold decrease in H3K4me3 at the promoter of *ESR1* (Fig. 5C).

We also analysed the H3K9me3 mark as a proxy for silencing, as a decrease in the occupancy of this mark would lead to enhanced gene expression [34]. In addition, this mark is particularly vulnerable to BPA exposure, and alterations in H3K9me3 could be inherited; e.g., BPA exposure causes a reduction in the repressive marks H3K9me3 and H3K27me3 in whole worms and germline nuclei in third-generation offspring [35]. To this end, we performed chromatin immunoprecipitation against H3K9me3 in cells exposed or not exposed to BPA. qPCR analysis was then performed for selected targets. In agreement with our immunofluorescence data (Fig. 3C, D), the analysis of individual genes showed a global tendency towards a decrease in H3K9me3 occupancy following BPA treatment. This H3K9me3 decrease was especially significant for the *STAT2* gene and the 5’UTR of LINE1 and slightly less significant for the SAT2 and SATA loci and PGR loci (Fig. 5D). We also detected decreases in H3K9me3 at the promoters of genes encoding factors involved in telomere function or maintenance, with *SMG7* and *XRCC5* being the most significantly affected (Fig. 5E). Thus, our data show that BPA treatment leads to decreased H3K9me3 occupancy at a large number of loci, suggesting that modifications of H3K9me3 occupancy could play a pivotal role in gene expression dysregulation following BPA exposure.

To reveal whether there was a change in DNA methylation, we extracted DNA from exposed and unexposed HeLa cells to BPA and performed MEDIP analysis at targets analysed in human blood. We observed an overall decrease in DNA methylation, although only a few genes showed a statically significant alteration, such as *ARID2, UPF2,* and a trend toward a decrease in *PGR, BRCA1, ESR2, VEGF, LINE1* and *IGF2* (Additional file 1: Fig. S3).

Overall, our analysis of H3K4me3 and DNA methylation showed similar changes in BPA-exposed human blood and HeLa cells.

### ESR1/ERα binding to its targets

**Fig. 6:**
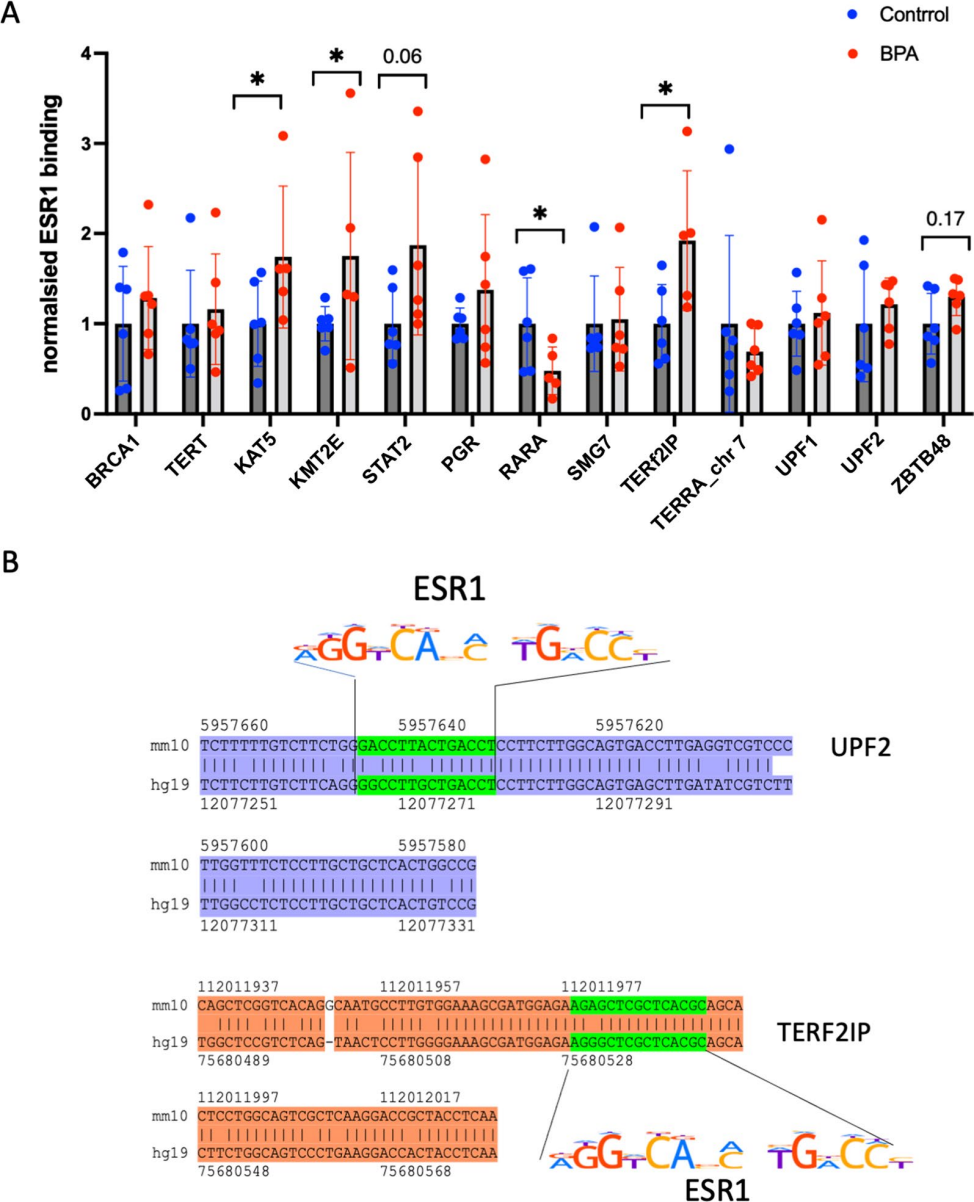
ESR1 binding analysis. **A** ESR1 ChIP‒qPCR analysis of ESR1 target genes; differential peak regions and those with ESR1 binding motifs were chosen for the analysis. *p < 0.05, **p < 0.01, Mann‒Whitney test, *n* = 6 experiments for each group. **B** ESR1 motif conservation analysis in the promoters of *UPF2* and *TERF2IP*

To test whether ESR1/ERα could be involved in the BPA-induced dysregulation of specific genes by directly binding to the promoters of *KAT5*, *RARA* and some telomere-associated genes, chromatin immunoprecipitation using anti-ESR1 antibody was performed, along with qPCR analysis of the targets. The design of the ChIP‒qPCR primers is detailed in the Methods section. The analysis showed a 1.7-, 1.8- and 1.9-fold increase in ESR1 binding at the *KAT5, KMT2E* and *STAT2* loci, respectively (Fig. 6A). For the five telomeric DNA-binding genes, an increase in ESR1 binding was identified only for *TERF2IP* (1.9-fold), suggesting that the alterations in telomeric DNA binding factor expression could be mediated by other factors.

To further reveal whether at least some gene promoters located in differential peaks have evolutionarily conserved ESR1 binding motifs, we used the ECR browser tool [36]. This tool is designed to highlight candidate functional elements in the genome by comparing sequences from several species and finding conserved blocks. Notably, both *UPF2* and *TERF2IP* gene regulatory regions were found to contain an ESR1 binding motif that was conserved between the mouse and human reference genome sequences (Fig. 6B), suggesting that these could be functional elements in both species.

### Analysis of ESR1 and p53/TP53 proteins in cells

**Fig. 7:**
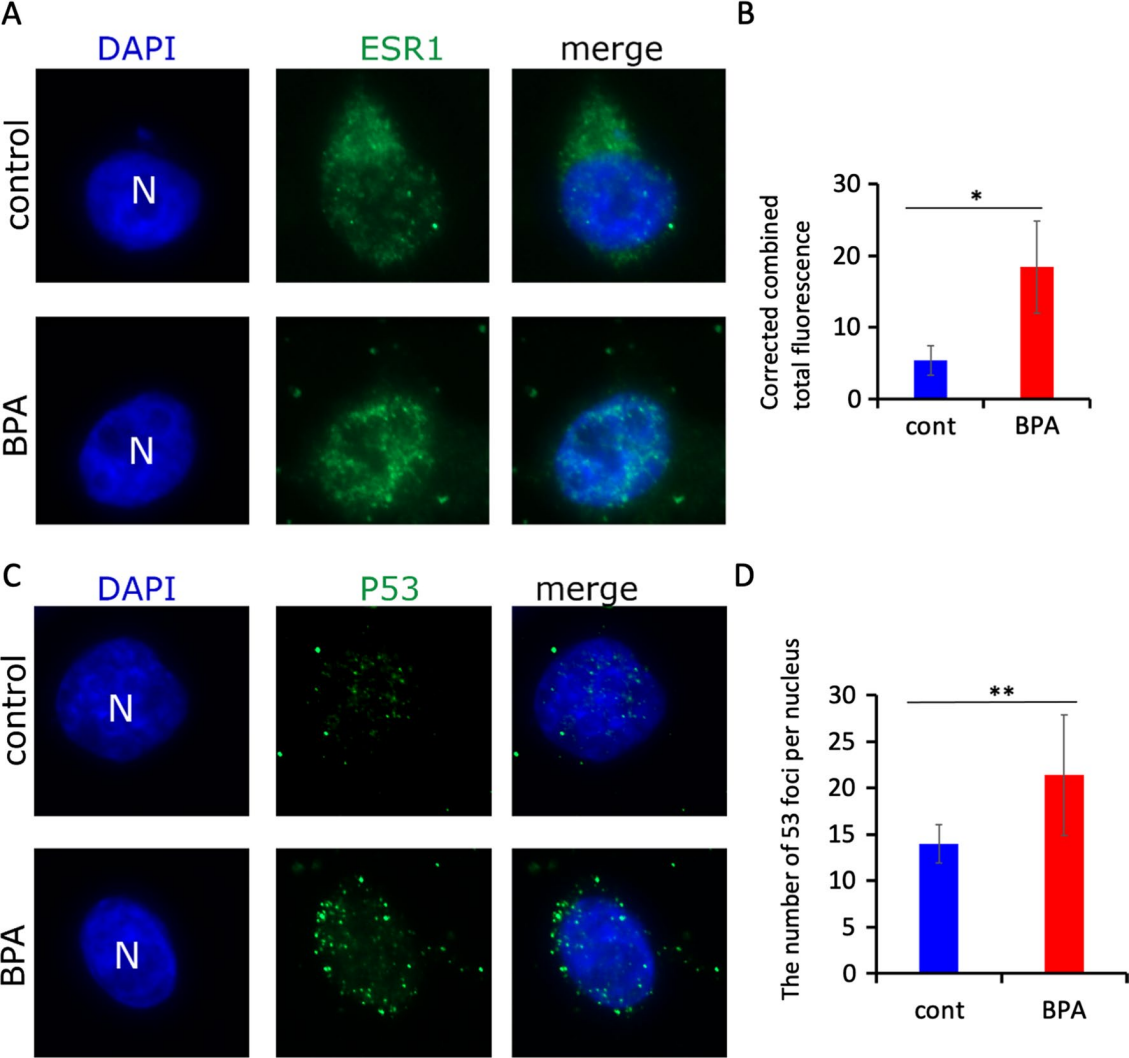
ESR1 and p53 immunofluorescence analysis in HeLa cells. **A** ESR1 staining in control cells (top) and in BPA-exposed cells (bottom); **B** Quantitative analysis of ESR1 fluorescence intensity; **C** p53 localisation in control cells (top) and in BPA-exposed cells (bottom); **D** Quantitative analysis of p53 foci per nucleus

Our analysis in both human blood and HeLa cells revealed that some of the ESR1 pathway targets were altered at epigenetic and transcriptional levels, we did not observe any changes in the ESR1 gene (which encodes ESR1/ERα) expression. We hypothesized that the observed changes seen in ESR1-target genes expression could be mediated by the changes in ESR1/ERα protein levels. We therefore performed analysis in HeLa cells with preserved nuclei and z-stack analysis of cells immunolabeled with an anti-ESR1 antibody. ESR1 staining appeared as a dot in both the nucleus and the cytoplasm (Fig. 7A); in exposed cells, the staining was mostly nuclear (Fig. 7A). Quantitative analysis revealed that there was a 3.4-fold increase in nuclear ESR1 staining in exposed cells compared to unexposed cells (Fig. 7B), suggesting that there is activation of ESR1 at the protein level.

Thus, our data suggest that BPA exposure probably stabilizes the ERa protein.

Since BPA exposure could lead to genotoxic stress, we decided to check whether cell exposure to 10 nM BPA could induce p53, a key player of the DNA damage response pathway. Using an immunoassay against p53, we observed that p53 also appeared as a focus, localised in both the nucleus and the cytoplasm (Fig. 7C). Quantitative analysis of foci showed an increased amount of p53 in exposed cells (Fig. 7D), mainly in the nucleus, 1.5-fold higher than that observed in unexposed cells (Fig. 7D).

Our immunofluorescence data therefore show that ESR1 and p53 localise more in the nucleus in treated cells than in unexposed cells.

## Discussion

### Effects of BPA exposure on genetic stability

An impact on the normalized DNA repeat number variations (Fig. 1) in alpha-satellite DNA was observed, mainly as an overall increase in averaged values in the high BPA group compared to the low BPA group. Alpha-satellite DNA is the main component of functional centromeres and is the main structural constituent of pericentromeric heterochromatin [37]. Satellite DNA consists of very large arrays of tandemly repeated DNA. We analysed human satellite II DNA (SAT2), which is found in pericentromeric heterochromatin and is most abundant on chromosome 1 (1qh) [38]. The centromeric satellite α DNA (SATA), located on chromosome 4 [39], was also assessed. Moreover, PCR-based profiling of centromeres revealed the heterogeneity of centromeric and pericentromeric sequences in cancer cells and tissues [40].

Satellite sequences in centromeric and pericentromeric regions are considered to be silent; however, they are actively transcribed in several biological processes, including cancer [41, 42]. In the blood of metastatic cancer patients, significantly increased levels of intracellular alpha satellite RNA were detected [43]. Some authors have suggested that aberrant expression of satellite RNAs accelerates oncogenesis through mechanisms involving increased DNA damage [44]. Therefore, it is conceivable that the increase in SATA repeat number caused by BPA could be involved in increased DNA damage. This could be therefore one of the mechanisms involved in the genotoxic effects of this environmental pollutant.

We also observed a reduction of DNA methylation at LINE1 promoters, and assume that this reduction leads to an increase in the gene expression. However, we did not analyse the LINE1 expression. Further work should address this question. As already discussed, BPA could induce the genotoxic stress through the generation of ROS. In this regard, we observed increased expression of DNA repair genes and p53 protein in the nucleus, suggesting a possible activation of the DNA damage pathway.

Thus, epigenetic alterations in DNA damage response genes, increase in p53 foci and alterations in satellite repeats number represent strong clues that BPA exposure may have genotoxic consequences.

### Changes in the epigenetic landscape in BPA-exposed adolescent males

In this work, we also examined the potential impact of BPA on DNA methylation at the promoters of ESR1-regulating genes. Adolescents in the high BPA group tended to have lower DNA methylation, with more evident effects on the methylation of imprinted genes and LINE1 elements. Changes in global DNA methylation were previously observed in BPA-exposed mice, where DNA methylation at the imprinted genes *Igf2r, Peg3* and *H19* decreased with increasing BPA concentrations [45]. These data are consistent with our results (Fig. 1B). Notably, altered *IGF2* expression has been observed in metabolic conditions such as obesity, diabetes and polycystic ovary syndrome [46]. Moreover, in mice, BPA exposure disrupts metabolic health across multiple generations [47]. Our data are consistent with the observation that BPA impairs insulin signalling, which could perturb glucose homeostasis [48].

Strikingly, we observed a global increase in transcription in HeLa cells and an increase in histone H3K4me3 associated with transcription initiation. It is conceivable that ESR1/ERa stabilization by BPA could modify the epigenetic landscape of chromatin, leading to the unprogrammed expression of a large number of genes. Thus, the transcription-activating properties of BPA at the genome level would be a consequence of chromatin accessibility alterations. In both, human blood and HeLa cell line analysis, we confirmed that BPA exposure leads to increase in histone H3K4me3 occupancy and decrease in repressive DNA methylation.

Strikingly, we found that the regions showing differential H3K4me3 occupancy included several telomere binding proteins. Telomeres are known regions of instability, as many DNA repair factors are detected at telomeres [49, 50]. Specifically, we found that both the H3K4me3 level and gene expression were increased for the telomere protein-encoding gene *TERF2IP* (also known as RAP1). Human TERF2IP protects telomeric DNA from NHEJ [51, 52]. The NHEJ process at telomeres could lead to chromosome fusions. Loss of *TERF2IP* induces telomere recombination in the absence of NHEJ or a DNA damage signal [53]. In the samples of adolescent exposed to BPA, we observed an increased TL, although not statistically significant (FC = 1.15, *p* = 0.08). A previous study found a similar increase in relation to exposure to another xenoestrogen, chlordecone [54]. In addition, in this study, we also observed a decrease in *TERT* DNA methylation in adolescent samples. Several studies have showed that *TERT* has several ESR1 binding sites, and that ESR1 could activates *TERT*. For example, telomerase activity and mRNA expression of the catalytic subunit of telomerase (hTERT) were upregulated following oestradiol cell treatment in in vitro experiments [55]. Down-regulation of the *ESR1* gene (oestrogen receptor-alpha), but not *ESR2* (ER beta), inhibited oestrogen-stimulated telomerase function [56]. These data suggest that the telomere could be affected by epigenetic alteration of telomere factors and also due to oestrogenic properties of BPA to activate *TERT*.

In addition to telomere binding proteins, other DNA repair factors exhibited increased histone H3K4me3 levels, e.g., *ATM, ATR, BLM, FANCA, MDC1, MLH1, MSH2, MSH3* and *RAD51.* Thus, it is tempting to speculate that BPA-induced unprogrammed expression of genome maintenance genes leads to genetic instability at repetitive sequences, as illustrated by the Alu and SATA repeat number increases in the blood of highly exposed individuals exposed to higher BPA concentrations.

### Effects of BPA on HeLa cells

The HeLa cell line is derived from cervical cancer cells. These cells have been used in a large number of cancer studies and in many sex steroid hormone studies, for example, to evaluate the in vitro effects of oestradiol and oestrogen-like compounds [57–59]. We used 10 nM BPA, which is relevant to environmental exposures [60]. In HeLa cells exposed to BPA, we observed effects similar to those seen in our human samples, such as the activation of DNA repair factors. Notably, we detected a strong increase in *ARID2* expression. ARID2 is a component of the SWI/SNF complex, which is involved in nucleosome positioning to modulate gene expression and DNA repair [61]. It was shown that knockout of *ARID2* could contribute to disruption of DNA repair processes, presumably by blocking nucleotide excision repair (NER) [62]. The strong increase in *ARID2* expression upon BPA treatment in HeLa cells could be explained by an increase in DNA repair activity caused by BPA-induced DNA damage. In in vitro experiments with a similar dose of BPA on MCF10A and 184A1 cells, the authors reported an increase in the production of reactive oxygen species (ROS) as well as the activation of DNA repair factors, suggesting that a low dose of BPA can induce a genotoxic effect on cells in vitro [63].

On the other hand, we observed a significant increase in the expression of *TEN1,* which encodes a subunit of the CST complex. The CST complex (CTC1-STN1-TEN1) is vital for telomere maintenance. In addition to its role in telomere function, CST inhibits MRE11 binding to reversed forks, suggesting that the CST complex serves as an important fork protector during replication perturbation [64]. Thus, the observed changes in the gene expression of telomere-associated genes suggest that BPA exposure affects telomere stability and may perturb replication. Since humans are continuously exposed to BPA, these dysregulations could be particularly deleterious during embryogenesis and youth, when replication is very high.

Based on the results found in this work, a new mechanism of action of BPA (Additional file 1: Fig. S4) is proposed. BPA stabilizes ERa, which in turn activates histone acetyltransferases and methyltransferases and thereby increases access to chromatin. BPA-induced acetyltransferase and methyltransferase overexpression also alters the epigenetic landscape of DNA repair genes and telomere maintenance genes, which would lead to genetic instability. Thus, exposure to environmentally relevant doses of BPA leads to a global increase in chromatin accessibility that could result in stable alteration of expression for a large number of genes.

### Limitations of this study

The availability of human material was the main limiting factor, as well as the small number of subjects included, which reduced our ability to detect other possible effects of BPA. For example, we were not able to acquire sufficient material for the analysis of gene expression or protein levels in vivo. In addition, classification of subjects was performed on the basis of urinary BPA levels assessed in a single urine sample, which may lead to misclassification of exposure given the nonpersistent nature and short-term viability of this chemical compound. Moreover, BPA exposure was measured in samples collected before (when boys were between 9 and 10 yrs.) and the genetic markers were assessed, when the boys were between 15 and 17 years old. Notwithstanding these limitations, this dataset was sufficient to reveal some interrelated associations consistent with the hypothesized toxicological pathway. Another limitation is that the study included only males, and sex-dependent associations could not be tested. In addition, genetic polymorphism could contribute to the low statistical power of the observed results. However, DNA methylation is known to constitute a very stable “omics” signature over time [65]. Maternal smoking during pregnancy was associated with DNA methylation changes at 18 loci in the blood of children at a mean age of 8.1 years [66], suggesting that epigenetic changes could be persistent.

In addition to BPA, other environmental chemicals studied in the same cohort are also known to contribute to health issues [67–69], and future work should consider the influence of human real chemical mixture exposure.

## Materials and methods

### The INMA-Granada cohort

The Environment and Childhood (INMA)-Granada cohort began with the recruitment of 668 mother-male child pairs between 2000 and 2002 at the Hospital Universitario San Cecilio (HUSC) (Granada, Spain) to evaluate the relationship between prenatal exposure to endocrine-disrupting chemicals and male urogenital malformations [70]. Different follow-ups of the initial cohort have since been performed, specifically, when the children were 4–5 yrs (2005–2006, *n* = 220), 9–11 yrs (2010–2012, *n* = 300) and 15–17 yrs (2017–2019; *n* = 155) of age [71–73]. The principles of the Helsinki Declaration were followed at all times, and both the initial study and all follow-up studies were approved by the Granada Biomedical Research Ethics Committee.

Children provided a single nonfasting spot urine sample at the 9–11-year visit, and total BPA (free plus conjugated) was determined by liquid chromatography‒mass spectrometry as previously described in detail [74]. The median and interquartile range (IQR) BPA concentrations were 4.74 (2.86, 8.96) μg/L, respectively [75]. At the 15–17-year follow-up, peripheral venous blood was collected from the adolescents under nonfasting conditions. Whole-blood aliquots were stored at − 80 °C and sent on dry ice to the Institut de Recherche en Santé, Environnement et Travail - INSERM UMR1085), Rennes, France.

### Experimental design

A graphical overview of the experiments performed in this study is provided in Additional file 1: Fig. S1. Subjects were categorized into two groups: those with urinary BPA concentrations below the median value (*n* = 16; low BPA group) and those with urinary BPA values above the median value (*n* = 15; high BPA group). Histone H3K4me3 occupancy at the genome-wide level, satellite repeat number variations (CNVs) and DNA methylation changes were analysed in genomic DNA extracted from the blood samples. Genome-wide H3K4me3 occupancy was evaluated in 10 subjects in the low-BPA group and in 7 in the high-exposure group. We performed the analysis in samples for which the quantity of whole blood was sufficient for next-generation sequencing library preparation. Human blood studies were complemented with analysis of the effects of in vitro BPA exposure in HeLa cells. In these cells, global histone H4 acetylation as well as H3K9me3 levels, gene expression, DNA methylation, H3K4me3 occupancy and ESR1 binding were evaluated in BPA-exposed and unexposed cells.

### HeLa cell culture and treatment

HeLa cells are an immortal cancerous human cell line derived from cervical cancer cells. The cells were cultured in Gibco Dulbecco’s modified Eagle’s medium (DMEM) supplemented with 10% foetal calf serum. Cells were seeded in a 12- or 24-well plate (1.5·10^5^ cells/ml) and treated for 24 h with 10 nM BPA in vehicle (DMSO). Control samples were treated with vehicle only. Cells were harvested by trypsinization. For RNA or ChIP analysis, cells were pelleted and stored at − 80 °C.

### DNA extraction

DNA extraction was performed using DNeasy Blood & Tissue kits (Qiagen), following the manufacturer’s instructions. The DNA extraction protocol included an RNase A treatment step to eliminate contaminating RNA. The concentration of the DNA was measured using the QuantiFluor dsDNA system (Promega). The quality of the DNA was assessed by running samples on a 0.7% agarose gel; a homogenous high-molecular-weight signal was observed for each sample. The quality of the DNA was also confirmed by measurement of the A260 to A280 ratio, which was greater than 1.8 in all cases.

### Methylated DNA precipitation (MEDIP)

For DNA methylation analysis in human blood, the EpiMark Methylated DNA Enrichment Kit (NEB, #E2600S) was used. A total of 700 ng of DNA was sonicated using a Qsonica sonicator with the following parameters: efficiency, 60%; total sonication time, 6 min, 20 s “on” and 20 s “off”. The sonicated methylated DNA was precipitated using MBD2a-protein A-coated beads, the methylated DNA-MBD2a-protein A-coated bead complex was washed, and the DNA was eluted with elution buffer. The DNA concentration was determined according to the level of fluorescence produced by a dsDNA-binding dye (Promega). Then, 5–10 ng of methylated DNA was recovered following precipitation, and the unprecipitated sonicated starting material was used as the input. Equal amounts of methylated DNA and input were taken for qPCR using primers (Additional file 1: Table S7) located in the CpG island of each tested gene. A region in the *RPLP0* gene was used for background normalization. A nonparametric Mann–Whitney test was used to assess the statistical significance.

### qPCR analysis of repeat number variation experiments

The extracted genomic DNA was diluted to obtain a 0.2 ng/μL concentration, and equal amounts of extracted genomic DNA were used for qPCR. Quantitative PCR was performed using a 384-well plate. Each well contained 5 μL of SYBR® Green Master Mix (Bio-Rad), 0.05 μL for each primer pair (100 µM stock solution), 0.9 μL of H_2_O, and 4 μL (0.8 ng) of DNA template. qPCR was performed using a CFX 384 Real-Time System (Bio-Rad) with a two-step protocol: initial denaturation at 98 °C for 30 s followed by a 40-cycle amplification program of 98 °C for 15 s and 65 for 1 min. The melting program was applied after the PCR program. Normalized expression values were calculated with the CFX Manager program using *RLP0* as the reference gene. The primers used in this work are listed in Additional file 1: Table S7.

### Chromatin immunoprecipitation (ChIP)

We performed ChIP using rabbit polyclonal antibodies against H3K4me3 (Millipore, 07-473), H3K9me3 (Abcam), ESR1 (ab32063, Abcam). Equal amounts of material (~ 100 µL of human total blood or cells from one confluent cell well of a 6-well plate) were incubated in 1% paraformaldehyde solution for 10 min to crosslink proteins to DNA. Then, 100 μL of 1.25 M glycine was added to each sample to quench the unbound paraformaldehyde. Samples were centrifuged at 1000 rpm for 10 min at 4 °C, and the pellets containing cells were resuspended in 300 μL of SDS lysis buffer (1% (wt/vol) SDS, 10 mM EDTA, 50 mM Tris–HCl pH 8) in the presence of protease inhibitors. Chromatin was sonicated in SDS lysis buffer at 60% amplitude for 8 min (20 s on, 20 s off in a Qsonica 700 sonicator supplied with cup horn 431C2); these parameters allowed us to obtain ~ 400 bp fragments. After sonication, the samples were centrifuged at 12,800 rpm for 10 min at 4 °C, and the supernatant containing sonicated chromatin was transferred to a new container and diluted in 1.7 mL of the following buffer: 1.1% (vol/vol) Triton X-100, 1.2 mM EDTA, 16.7 mM Tris–HCl, 167 mM NaCl. A solution containing 20 μL of Dynabeads (10002D, Invitrogen) and 0.5 µL of target-specific antibody was added to the sample tubes and incubated overnight at 4 °C. Before adding the antibody and Dynabeads, 10 μL of each sample was collected as an “input sample” (starting material).

After overnight incubation with antibody-coated Dynabeads, the beads were washed for 5 min in each of the following four buffers, in sequence: (1) Low-salt buffer: 0.1% (wt/vol) SDS, 1% (vol/vol) Triton X-100, 2 mM EDTA, 20 mM Tris–HCl, 150 mM NaCl; (2) high-salt buffer: 0.1% (wt/vol) SDS, 1% (vol/vol) Triton X-100, 2 mM EDTA, 20 mM Tris–HCl pH 8, 500 mM NaCl; (3) LiCl buffer: 0.25 M LiCl, 1% (vol/vol) IGEPAL, 1 mM EDTA, 10 mM Tris–HCl, pH 8, 1% (wt/vol) deoxycholic acid; (4) TE buffer (two washes).

After the washing steps, the beads were treated two times with a 50 μL solution containing 1% (wt/vol) SDS, 0.1 M NaHCO_3_ pH 9 and incubated at 65 °C for 15 min to elute the precipitated chromatin from the beads. Subsequently, the eluted chromatin was reverse cross-linked by adding 9 μL of 5 M NaCl and incubating at 65 °C for 4 h. Then, proteins were removed by adding 40 µg of proteinase K and incubating the samples for 1 h at 45 °C. The precipitated DNA was purified with a MinElute Reaction Clean-Up kit (Qiagen), and the DNA concentration was measured using a QuantiFluor dsDNA system (Promega). A minimum of ~ 3 ng of DNA was recovered.

### ChIP–qPCR

A total of 0.4 ng of precipitated DNA or input sample was used for qPCR analysis (0.1 ng/μL). Quantitative PCR was performed as described before. Normalized expression values were calculated with the CFX Manager program using a region located far from the promoter as a reference gene, and we used a region in *RPLP0* for ChIP normalization. Enrichment of each target in the precipitated DNA was evaluated by calculating the ratio between the average of the normalized ChIP DNA copies and the average of the normalized DNA copies in the inputs. The primers used in this work are listed in Additional file 1: Table S7.

### Library preparation and ChIP-seq analysis

Sequencing libraries were prepared using the NEBNext Ultra DNA Library Prep Kit for Illumina (E7645S; NEB). We used 3–5 ng DNA for library preparation, and 15 cycles were used for library amplification. Sequencing was performed on an Illumina HiSeq4000 sequencer using a single-end 50-base read in multiplexed mode. Adapter dimer reads were removed using DimerRemover. Quality control of the reads was performed with FastQC v0.11.7. The sequencing read information is presented in Additional file 1: Table S8. The number of reads per sample is indicated. Reads were aligned to the human GRCh38 reference using Bowtie (v1.2.3) [76] (command line parameters: -m 1 --best --strata -v 3), sorted using SAMtools (v1.13) [77] and converted directly into binary files (BAM). PCR duplicated reads were marked and removed using SAMtools. For the visualization of ChIP-seq tracks, BedGraph tracks were generated using the GenomeCoverageBed function from SAMtools. IGV was used to visualize the tracks [78].

The regions with differential enrichment of H3K4me3 in the control and treatment were identified using the csaw (v1.26.0) package [79]. First, we counted count reads in the full genome using windowCounts() with spacing = 50, width = 150, ext = 530, shift = 0, filter = 10, and bin = FALSE parameters. Then, normalization factors were calculated using normFactors (). Then, estimateDisp () and glmQLFit () from the edgeR package (v3.34.0) [80] were used to calculate the log2-fold-change, *p* value and FDR of the differentially enriched regions. The significantly differentially enriched regions were selected at FDR < 0.05.

### Motif enrichment analysis

The repeat-masked sequences of regions with differential H3K4me3 enrichment were analysed. Motif identification was performed using MEME-ChIP [29] with the default parameters. The identified motifs were compared with known motifs using TomTom [81], and Find Individual Motif Occurrences (FIMO) was used to scan for motif-binding sites [82].

### Motif conservation analysis

We used the ECR browser tool [36]. The ECR Browser tool is designed to highlight candidate functional elements in the genome by comparing sequences of several species and finding conserved blocks.

### RNA extraction and quantitative PCR

Total RNA was extracted using an RNeasy Plus Mini Kit (Qiagen) according to the manufacturer’s instructions. This kit includes a DNA elimination step. Reverse transcription of 1 µg of RNA was performed using an iScript™ cDNA Synthesis Kit (Bio-Rad). The resulting cDNA was diluted 10 times and used for quantitative RT‒qPCR. The primer sequences used for RT‒qPCR are shown in Additional file 1: Table S9. RT‒qPCR was performed using iTaq Universal SYBR Green SuperMix (Bio-Rad) according to the manufacturer**’**s instructions on a CFX384 Touch Real-Time PCR Detection System (Bio-Rad). The quantification cycle (Cq) values were calculated using Bio-Rad CFX Manager 3.1. The Cq values of *RPL37A* cDNA were used for normalization, as this housekeeping gene showed low variation among all samples. The data were analysed and are presented as the mean fold change (FC) values compared to the control ± SD, *p < 0.05, **p < 0.01, ***p < 0.001.

### Immunofluorescence

For immunofluorescence, cells were fixed with 1% paraformaldehyde for 1 h, washed with PBS, additionally quenched with 10 nM glycine, permeabilized with 0.15% Triton X-100 and blocked with 4% BSA. Cells were incubated at 4 °C with either 1:500 anti-H4 hyperacetylated histone (06–956, Millipore) or 1:500 anti-H3K9me3 (ab8898, Abcam) or 1:200 anti-ESR1 (ab32063, Abcam), or 1:200 anti-p53 (ab1101, Abcam) primary antibody, washed, incubated with Alexa-conjugated secondary antibody for 45 min and mounted in Vectashield containing DAPI. The cells were then analysed using a Zeiss fluorescence microscope, and images were taken using AxioVision software. Images were obtained using an AxioImager microscope equipped with an AxioCam MRc5 camera and AxioVision software version 4.8.2 (Zeiss, Germany) with a 63X objective lens. Z-stacks were acquired with a 500 nm step; 15 individual planes were taken for each individual channel for DAPI and H3K9me3, ESR1, p53 or H4Ac using the Zen Pro (version 2.3) program. All images for control and exposed samples were taken with a fixed exposure time. Deconvolution was performed using the “Fast Iterative” algorithm provided by Zen Pro. A summed projection was generated for each z-stack, and the resulting images were analysed in ImageJ v1.52n. We used the lasso tool for nucleus and cytoplasm contouring, and the integrated immunofluorescence density for each nucleus was calculated. A similar area within the background was measured and subtracted from the fluorescent signal. The signal for the histone mark was then normalized to the DAPI signal. We analysed 4–5 independent biological replicates for the control and treated groups, with at least 15 cells for each replicate. The data were plotted in Excel and presented as normalized total cell fluorescence. We counted p53 foci in nucleus and the data is presented as averaged number of p53 foci per nucleus.

### Primer design for ChIP‒qPCR (for unique regions)

To design primers for ChIP‒qPCR analysis of regions found to have differential H3K4me3 occupancies, we identified the coordinates of altered regions using the Integrative Genomics Viewer genome browser and bed file results from MACS2 peak calling. We placed the coordinates of the peaks from the bed file into the UCSC genome browser to retrieve the genomic DNA sequences. We masked repeated sequences to obtain unique sequences to avoid multiple priming. We designed primers near the centre of the peaks (± 200 bp) by using the Primer-BLAST tool from the National Center for Biotechnology Information (NCBI).

### Statistical analysis

The Mann–Whitney test was used to evaluate the differences between the two subject groups as well as for all quantitative analyses in HeLa cells. The low BPA group (*n* = 16, BPA range: 0.66–4.67 µg/g) was compared to the high BPA group (*n* = 15, BPA range: 4.85–42.64 µg/g) for assessment in most of our analyses.

## Supplementary Information

**Additional file 1: Figure S1**. Schematic presentation of the experiments performed in this study in human samples and HeLa cells. **Figure S2**. Gene Ontology generated by GREAT. (A) Gene ontology “Biological process”, (B) Gene Ontology “Cellular component”. **Figure S3**. DNA methylation analysis at the promoters: (A) DNA repair genes, (B) oestrogen signalling pathway genes, (C) telomere maintenance and chromatin factors genes (D) of imprinted genes and repeat regions. *p < 0.05, Mann‒Whitney test. **Figure S4**. A proposed mechanism of BPA action. BPA stabilizes ESR1, which in turn activates histone acetyltransferases and methyltransferases and thereby increases access to chromatin. This complex activates the gene expression at several regions. In addition, the genotoxic effect of BPA induces the expression of telomere maintenance genes and DNA repair factors. **Table S1**. The human samples used for the DNA repeat analysis. **Table S2**. The human samples used for DNA methylation analysis. **Table S3**. The human samples used for Chip-seq analysis. **Table S4**. Genes encoding telomeric DNA binding proteins located in differential peaks. **Table S5**. Genes encoding ESR1-interacting proteins located in differential H3K4me3 peaks. **Table S6**. Genes encoding DNA repair proteins located in differential H3K4me3 peaks. **Table S7**. Primers used for repeat, Chip-qPCR and Medip-qPCR analysis. **Table S8**. ChIP-seq reads. **Table S9**. Primers used for RT-qPCR in this work.

## Data Availability

All sequencing and ChIP-seq data from this study are publicly available and have been deposited in the National Center for Biotechnology Information Gene Expression Omnibus. The datasets supporting the conclusions of this article are available in the [GEO] repository, [GSE212713] and hyperlink to datasets https://www.ncbi.nlm.nih.gov/geo/query/acc.cgi?acc=GSE212713.

## Abbreviations

AHR: Aryl hydrocarbon receptor
AR: Androgen receptor
ARID2: AT-rich interaction domain 2
ATM: ATM serine/threonine kinase
BDNF: Brain derived neurotrophic factor
BLM: BLM RecQ like helicase
BPA: Bisphenol A
BRCA1: BRCA1 DNA repair associated
RNV: Repeat number variation
CTC1: CST telomere replication complex component 1
DAVID: The Database for Annotation, Visualization and Integrated Discovery
ERE: Estrogen response element
ESRRA: Estrogen related receptor alpha
ESR1: Estrogen receptor 1
ESR2: Estrogen receptor 2
EZH2: Enhancer of zeste 2 polycomb repressive complex 2 subunit
FANCA: FA complementation group A
FIMO: Find individual motif occurrence
GREAT: Genomic Regions Enrichment of Annotations Tool
HBM4EU: Human Biomonitoring for Europian Union
IGF2: Insulin like growth factor (IGF2)
IGF2: Insulin like growth factor 2
KAT5: Lysine acetyltransferase 5
KCNQ1: Potassium voltage-gated channel subfamily Q member 1
KMT2E: Lysine methyltransferase 2E (inactive)
LINE1: Long interspersed nuclear element-1
MCF-7: Michigan Cancer Foundation-7
MDC1: Mediator of DNA damage checkpoint 1
MEDIP: Methylated DNA immunoprecipitation
MEME: Multiple EM for Motif Elicitation
MLH1: MutL homolog 1
MSH2: MutS homolog 2
MSH3: MutS homolog 3
NCOR1: Nuclear receptor corepressor 1
NCOR2: Nuclear receptor corepressor 2
NER: Nucleotide Excision Repair
NHEJ: Non-homologous end joining
NPCs: Neuronal progenitor cells
ORF2: Open reading frame 2
PGR: Progesterone receptor
PPI: Protein–Protein Interaction
PURA: Purine rich element binding protein A
RAD51: RAD51 recombinase
RARA: Retinoic acid receptor alpha
ROS: Reactive oxygen species
RPA1: Replication protein A1
RPL37A: Ribosomal protein L37a
RXRA: Retinoid X receptor alpha
SATA: Satellite alpha
SINEB1: Short Interspersed Nuclear Element B1
SMG7: SMG7 nonsense mediated mRNA decay factor
STAT2: Signal transducer and activator of transcription 2
STN1: STN1 subunit of CST complex
SUV39H1: SUV39H1 histone lysine methyltransferase
SUV39H2: SUV39H2 histone lysine methyltransferase
TL: Telomere length
TEN1: TEN1 subunit of CST complex
TERF2IP: TERF2 interacting protein
TERRA: TElomeric Repeat-containing RNA
TERT: Telomerase reverse transcriptase
TP53: Tumor protein p53
UPF1: UPF1 RNA helicase and ATPase
UPF2: UPF2 regulator of nonsense mediated mRNA decay
VEGFA: Vascular endothelial growth factor A
ZBTB48: Zinc finger and BTB domain containing 48
