# Supplementary material for: Genome-wide distribution of histone trimethylation reveals a global impact of bisphenol A on telomeric binding proteins and histone acetyltransferase factors: a pilot study with human and in vitro data
Source: Clin Epigenetics. 2022 Dec 26;14:186. doi: 10.1186/s13148-022-01408-2 (PMC9793539; doi:10.1186/s13148-022-01408-2)
Supplement: Supplementary file 1 — Additional file 1: Figure S1. Schematic presentation of the experiments performed in this study in human samples and HeLa cells. Figure S2. Gene Ontology generated by GREAT. (A) Gene ontology “Biological process”, (B) Gene Ontology “Cellular component”. Figure S3. DNA methylation analysis at the promoters: (A) DNA repair genes, (B) oestrogen signalling pathway genes, (C) telomere maintenance and chromatin factors genes (D) of imprinted genes and repeat regions. *p < 0.05, Mann‒Whitney test. Figure S4. A proposed mechanism of BPA action. BPA stabilizes ESR1, which in turn activates histone acetyltransferases and methyltransferases and thereby increases access to chromatin. This complex activates the gene expression at several regions. In addition, the genotoxic effect of BPA induces the expression of telomere maintenance genes and DNA repair factors. Table S1. The human samples used for the DNA repeat analysis. Table S2. The human samples used for DNA methylation analysis. Table S3. The human samples used for Chip-seq analysis. Table S4. Genes encoding telomeric DNA binding proteins located in differential peaks. Table S5. Genes encoding ESR1-interacting proteins located in differential H3K4me3 peaks. Table S6. Genes encoding DNA repair proteins located in differential H3K4me3 peaks. Table S7. Primers used for repeat, Chip-qPCR and Medip-qPCR analysis. Table S8. ChIP-seq reads. Table S9. Primers used for RT-qPCR in this work. [file 13148_2022_1408_MOESM1_ESM.pdf]

Shereen Cynthia D'Cruz*[1], Chunxiang Hao*[2], Martin Labussiere1, Vicente Mustieles[3,4,5], Carmen Freire[3,4,5,] Louis Legoff[1], Laura Magnaghi-Jaulin[1], Alicia Olivas-Martinez[3,4], Andrea Rodriguez-Carrillo[3,4], Christian Jaulin[1], Arthur David[1], Mariana F. Fernandez[3,4,5]≠ and Fatima Smagulova[1]≠

1 Univ. Rennes, EHESP, Inserm, Irset (Institut de recherche en santé, environnement et travail) - UMR_S 1085, F-35000, Rennes, France

2 School of Medicine, Linyi University, Linyi 276000, China

3 Center for Biomedical Research (CIBM) & Department of Radiology and Physical Medicine, School of Medicine, University of Granada, 18016, Granada, Spain

4 Instituto de Investigación Biosanitaria (ibs.GRANADA), 18012 Granada, Spain

5 Consortium for Biomedical Research in Epidemiology & Public Health (CIBERESP), 28029 Madrid, Spain

* equal contribution

# Genome-wide distribution of histone trimethylation reveals a global impact of bisphenol A on telomeric binding proteins and histone acetyltransferase factors: A pilot study with human and *in vitro* data.

Supplementary information

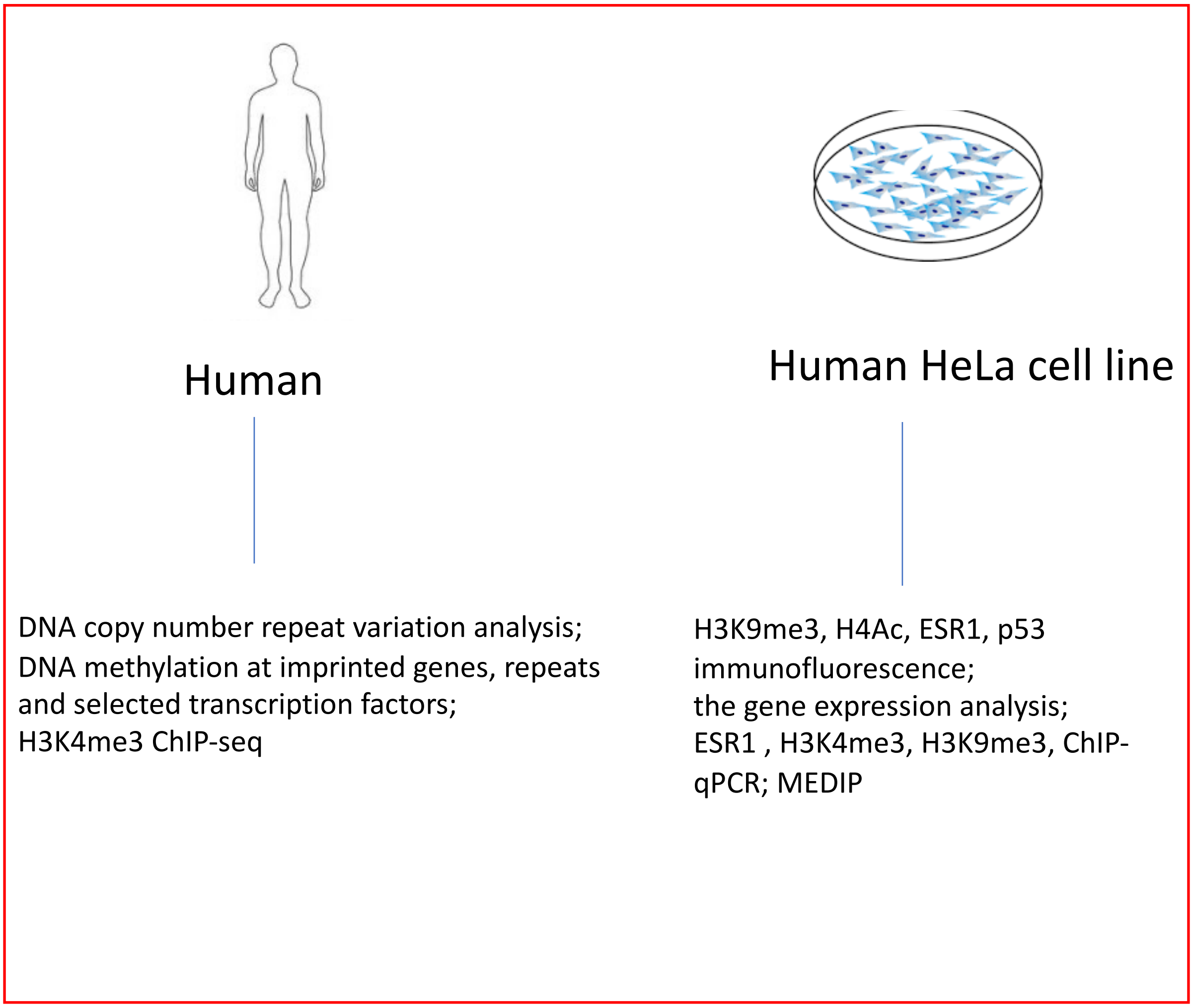

Figure S1. Schematic presentation of the experiments performed in this study in human samples and HeLa cells.

A

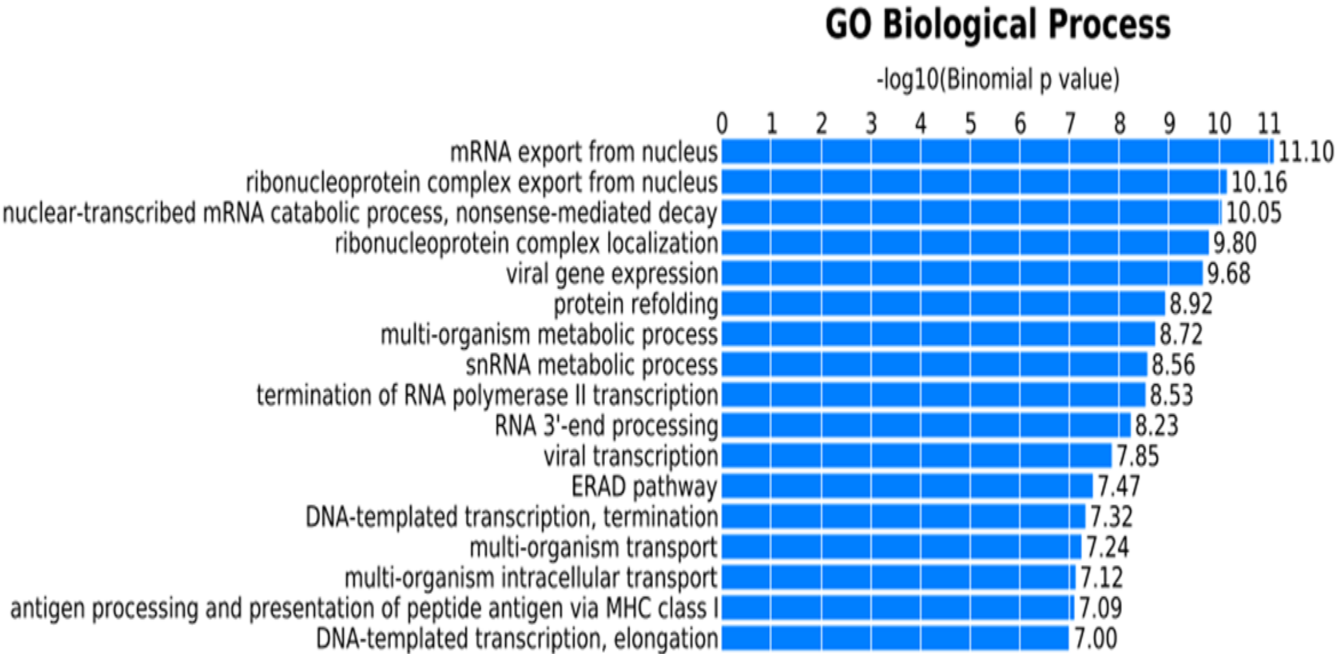

B

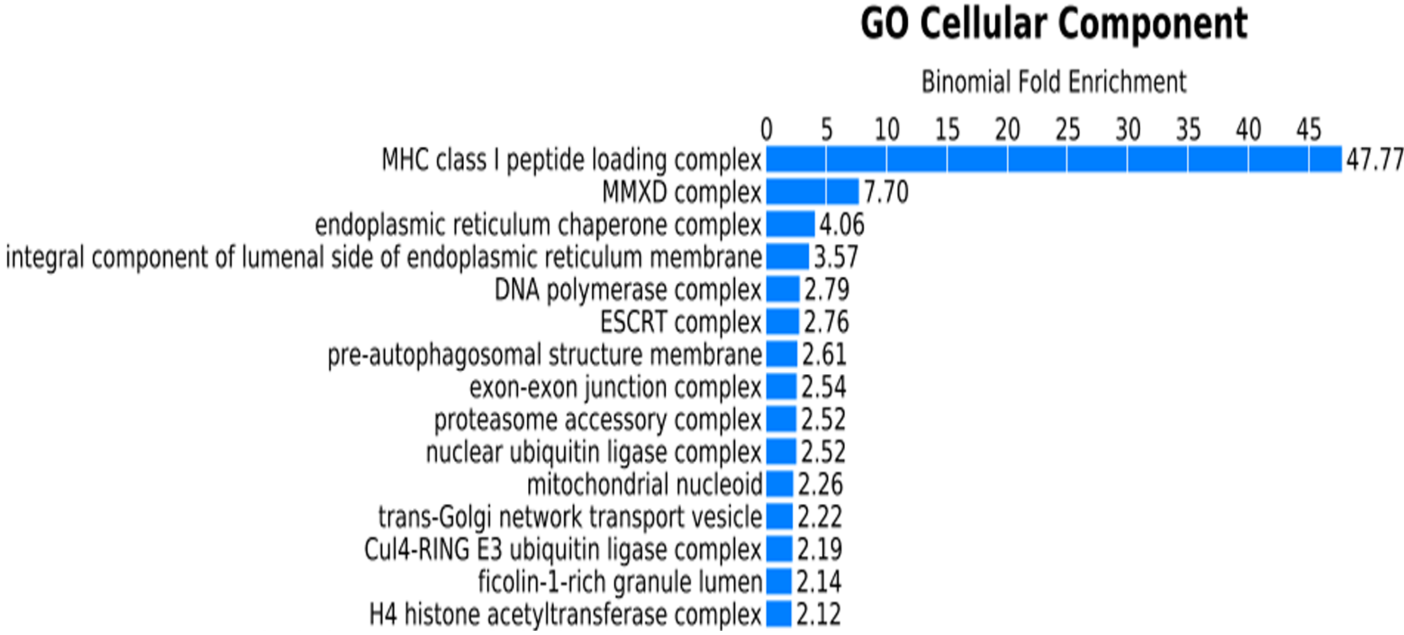

Figure S2. Gene Ontology generated by GREAT. A) Gene Ontology "Biological process", B) Gene Ontology "Cellular component".

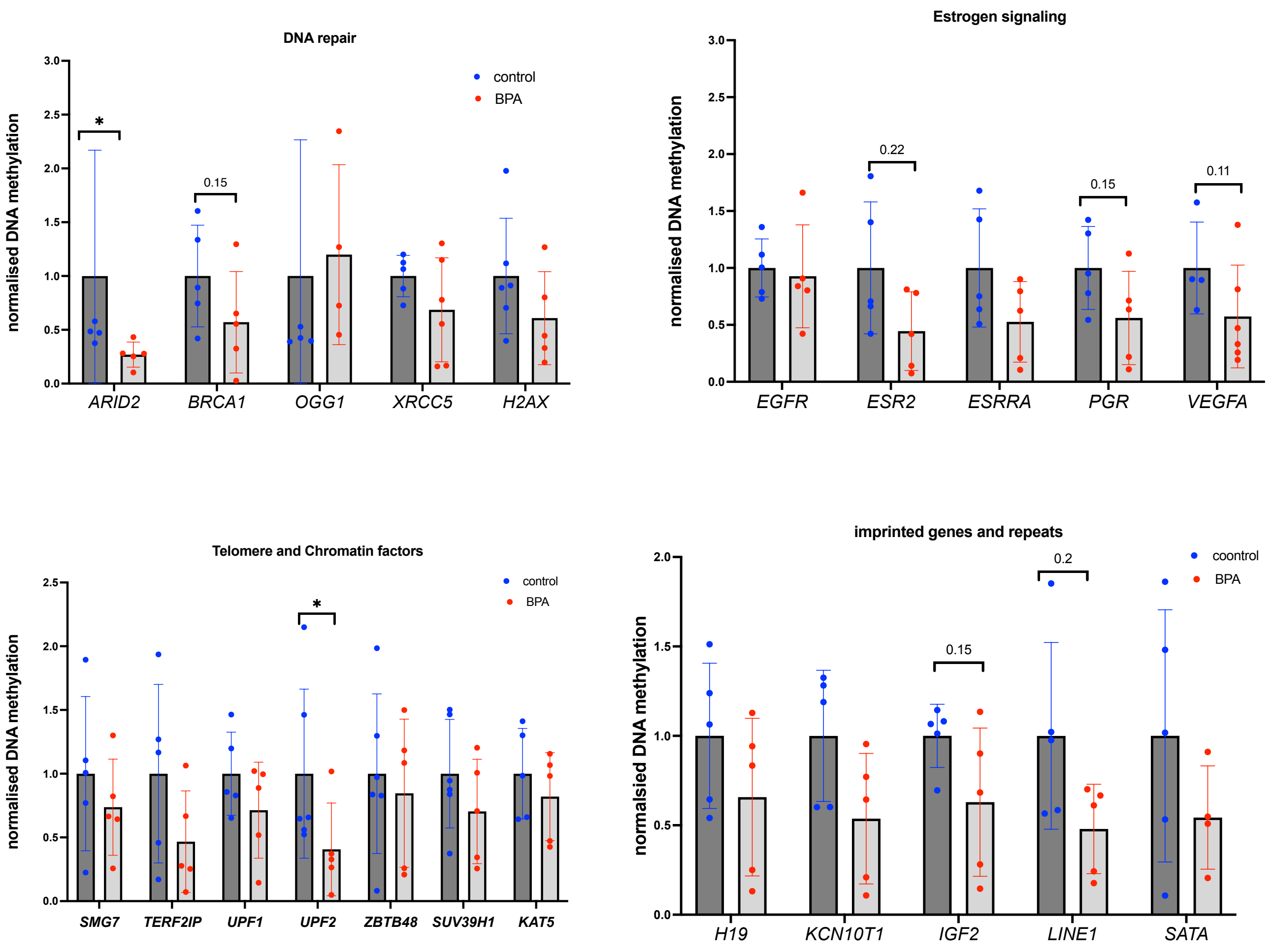

Figure S3. DNA methylation analysis at the promoters: A) DNA repair genes, B) oestrogen signalling pathway genes, C) telomere maintenance and chromatin factors genes D) of imprinted genes and repeat regions. *p<0.05, Mann–Whitney test.

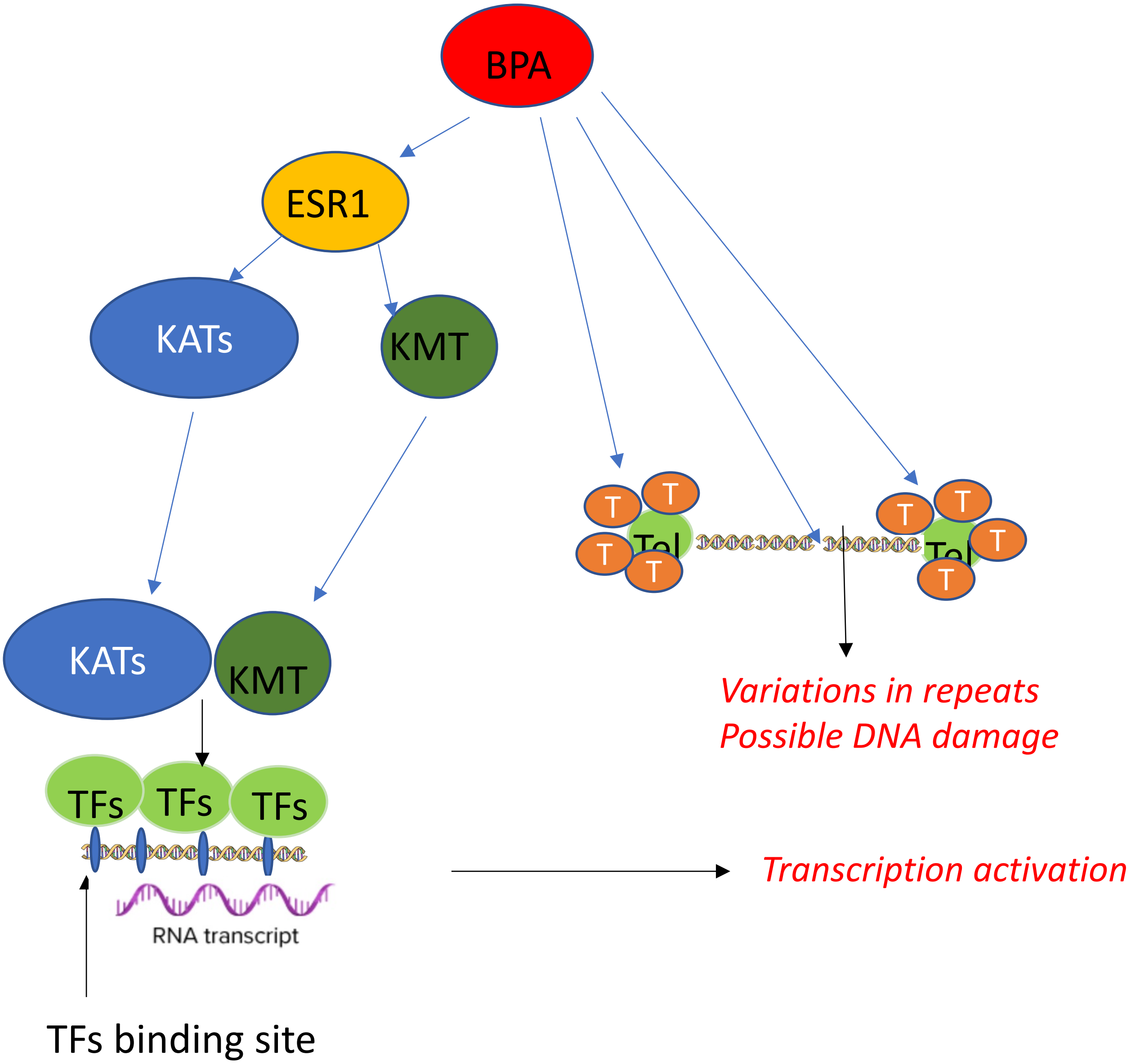

Figure S4. A proposed mechanism of BPA action. BPA stabilizes ESR1, which in turn activates histone acetyltransferases and methyltransferases and thereby increases access to chromatin. This complex activates the gene expression at several regions. In addition, the genotoxic effect of BPA induces the expression of telomere maintenance genes and DNA repair factors.

Table S1. The human samples used for the DNA repeat analysis

N	BPA (microg/gram Creat)	BPA gpoup
1	0,66	low
2	0,73	low
3	1,1	low
4	1,12	low
5	1,28	low
6	2,13	low
7	2,31	low
8	2,35	low
9	2,43	low
10	2,55	low
11	3,1	low
12	3,94	low
13	4,47	low
14	4,55	low
15	4,57	low
16	4,67	low
17	4,85	high
18	5,1	high
19	6,28	high
20	7,46	high
21	8,14	high
22	8,98	high
23	9,44	high
24	10,07	high
25	10,55	high
26	12,24	high
27	15,87	high
28	17,11	high
29	19,57	high
30	39,34	high
31	42,64	high

Note, Creat., creatinine, microg., mircrogram

Table S2. The human samples used for DNA methylation analysis

	BPA (microg/gram Creatinine)	BPA group
1	0,66	low
2	0,73	low
3	1,1	low
4	1,12	low
5	1,28	low
6	2,13	low
7	2,31	low
8	2,35	low
9	2,43	low
10	2,55	low
11	3,1	low
12	3,94	low
13	9,44	high
14	10,07	high
15	10,55	high
16	12,24	high
17	15,87	high
18	17,11	high
19	19,57	high
20	39,34	high
21	42,64	high

Note, Creat., creatinine, microg., mircrogram

Table S3. The human samples used for Chip-seq analysis

N	BPA (microg/gram Creat)	BPA gpoup
1	0,66	low
2	0,73	low
3	1,1	low
4	1,12	low
5	1,28	low
6	2,13	low
7	2,31	low
8	2,35	low
9	2,43	low
10	2,55	low
11	10,07	high
12	10,55	high
13	12,24	high
14	17,11	high
15	19,57	high
16	39,34	high
17	42,64	high

Note, Creat., creatinine, microg., mircrogram

Table S4. Genes encoding telomeric DNA binding proteins located in differential peaks

Gene	distance to TSS	description
ACD	(+344)	ACD shelterin complex subunit and telomerase recruitment factor
CTC1	(+25,553)	CST telomere replication complex component 1
HNRNPA2B1	(+95)	heterogeneous nuclear ribonucleoprotein A2/B1
HNRNPD	(+570)	heterogeneous nuclear ribonucleoprotein D
KDM1A	(+228), (+148,773)	lysine demethylase 1A
NABP2	(-2,573)	nucleic acid binding protein 2
NCL	(+178)	nucleolin
PURA	(-6,082), (+8)	purine rich element binding protein A
RPA1	(-43)	replication protein A1
SMG1	(+218)	SMG1 nonsense mediated mRNA decay associated PI3K related kinase
SMG5	(+149)	SMG5 nonsense mediated mRNA decay factor
SMG6	(+145)	SMG6 nonsense mediated mRNA decay factor
SMG7	(-527)	SMG7 nonsense mediated mRNA decay factor
TEN1	(+101)	TEN1 subunit of CST complex
TERF2IP	(+830)	TERF2 interacting protein
TERT	(+96)	telomerase reverse transcriptase
TP53BP1	(+190)	tumor protein p53 binding protein 1
UPF1	(+815)	UPF1 RNA helicase and ATPase
UPF2	(-6,794)	UPF2 regulator of nonsense mediated mRNA decay
UPF3A	(+282)	UPF3A regulator of nonsense mediated mRNA decay
XRCC6	(-234)	X-ray repair cross complementing 6
ZBTB48	(+288), (+21,528)	zinc finger and BTB domain containing 48

Table S5. Genes encoding ESR1-interacting proteins located in differential H3K4me3 peaks

	Gene	description
1	AHR	aryl hydrocarbon receptor
2	ESRRA	estrogen related receptor alpha
3	NCOR1	nuclear receptor corepressor 1
4	NCOR2	nuclear receptor corepressor 2
5	NR2F6	nuclear receptor subfamily 2 group F member 6
6	PSMD2	proteasome 26S subunit ubiquitin receptor, non-ATPase 2
7	RARA	retinoic acid receptor alpha
8	RXRA	retinoid X receptor alpha
9	TFRC	transferrin receptor
10	ABCC5	ATP binding cassette subfamily C member 5
11	ABCF1	ATP binding cassette subfamily F member 1
12	ABL1	ABL proto-oncogene 1, non-receptor tyrosine kinase
13	ACAA1	acetyl-CoA acyltransferase 1
14	ACLY	ATP citrate lyase
15	ACO2	aconitase 2
16	ACTB	actin beta
17	ACTN1	actinin alpha 1
18	ACTN4	actinin alpha 4
19	ACTR2	actin related protein 2
20	ACTR3	actin related protein 3
21	AHNAK	AHNAK nucleoprotein
22	AIFM1	apoptosis inducing factor mitochondria associated 1
23	AKAP13	A-kinase anchoring protein 13
24	AKAP8	A-kinase anchoring protein 8
25	AKT2	AKT serine/threonine kinase 2
26	ALDH18A1	aldehyde dehydrogenase 18 family member A1
27	ALDOA	aldolase, fructose-bisphosphate A
28	ALKBH7	alkB homolog 7
29	ANP32A	acidic nuclear phosphoprotein 32 family member A
30	ANP32B	acidic nuclear phosphoprotein 32 family member B
31	ANXA2	annexin A2
32	AP2M1	adaptor related protein complex 2 subunit mu 1
33	APOD	apolipoprotein D
34	ARF1	ADP ribosylation factor 1
35	ARFGAP2	ADP ribosylation factor GTPase activating protein 2
36	ARHGDIA	Rho GDP dissociation inhibitor alpha
37	ARID5A	AT-rich interaction domain 5A
38	ARNT	aryl hydrocarbon receptor nuclear translocator
39	ARPC4	actin related protein 2/3 complex subunit 4
40	ARSB	arylsulfatase B
41	ASF1A	anti-silencing function 1A histone chaperone
42	ASH2L	ASH2 like, histone lysine methyltransferase complex subunit
43	ASXL1	ASXL transcriptional regulator 1
44	ATAD2	ATPase family AAA domain containing 2
45	ATAD3A	ATPase family AAA domain containing 3A
46	ATF1	activating transcription factor 1
47	ATP1A1	ATPase Na+/K+ transporting subunit alpha 1
48	ATP2A2	ATPase sarcoplasmic/endoplasmic reticulum Ca2+ transporting 2
49	ATP2B1	ATPase plasma membrane Ca2+ transporting 1
50	ATP5B	None
51	ATP5F1	None
52	BAG1	BAG cochaperone 1
53	BARD1	BRCA1 associated RING domain 1
54	BAZ1B	bromodomain adjacent to zinc finger domain 1B
55	BCL3	BCL3 transcription coactivator
56	BCLAF1	BCL2 associated transcription factor 1
57	BLCAP	BLCAP apoptosis inducing factor
58	BTF3	basic transcription factor 3
59	BZW1	basic leucine zipper and W2 domains 1
60	CALM1	calmodulin 3
61	CALM1	calmodulin 1
62	CALM2	calmodulin 2
63	CALM2	calmodulin 3
64	CALM2	calmodulin 1
65	CALM3	calmodulin 3
66	CAND1	cullin associated and neddylation dissociated 1
67	CAPRIN1	cell cycle associated protein 1
68	CAPZB	capping actin protein of muscle Z-line subunit beta
69	CCDC110	coiled-coil domain containing 110
70	CCT7	chaperonin containing TCP1 subunit 7
71	CD47	CD47 molecule
72	CDC25B	cell division cycle 25B
73	CDK11B	cyclin dependent kinase 11B
74	CDK5	cyclin dependent kinase 5
75	CDK8	cyclin dependent kinase 8
76	CEBPA	CCAAT enhancer binding protein alpha
77	CEBPB	CCAAT enhancer binding protein beta
78	CFL1	cofilin 1
79	CHD3	chromodomain helicase DNA binding protein 3
80	CHD4	chromodomain helicase DNA binding protein 4
81	CHD9	chromodomain helicase DNA binding protein 9
82	CHUK	component of inhibitor of nuclear factor kappa B kinase complex
83	CLTC	clathrin heavy chain
84	CNDP2	carnosine dipeptidase 2
85	CNOT1	CCR4-NOT transcription complex subunit 1
86	COPA	COPI coat complex subunit alpha
87	COPB1	COPI coat complex subunit beta 1
88	COPS5	COP9 signalosome subunit 5
89	CSE1L	chromosome segregation 1 like
90	CSNK2A1	casein kinase 2 alpha 1
91	CSNK2B	casein kinase 2 beta
92	CSNK2B	novel protein
93	CTNNB1	catenin beta 1
94	CTSD	cathepsin D
95	CUL4B	cullin 4B
96	DAGLB	diacylglycerol lipase beta
97	DAP3	death associated protein 3
98	DBN1	drebrin 1
99	DDOST	dolichyl-diphosphooligosaccharide--protein glycosyltransferase non-catalytic subunit
100	DDX17	DEAD-box helicase 17
101	DDX18	DEAD-box helicase 18
102	DDX21	DExD-box helicase 21
103	DDX3X	DEAD-box helicase 3 X-linked
104	DDX5	DEAD-box helicase 5
105	DDX54	DEAD-box helicase 54
106	DECR1	2,4-dienoyl-CoA reductase 1
107	DEK	DEK proto-oncogene
108	DHX15	DEAH-box helicase 15
109	DHX30	DExH-box helicase 30
110	DHX9	DExH-box helicase 9
111	DKC1	dyskerin pseudouridine synthase 1
112	DNAJB11	DnaJ heat shock protein family (Hsp40) member B11
113	DNM1L	dynamin 1 like
114	DTD1	D-aminoacyl-tRNA deacylase 1
115	DYNC1H1	dynein cytoplasmic 1 heavy chain 1
116	EDF1	endothelial differentiation related factor 1
117	EEF1A1	eukaryotic translation elongation factor 1 alpha 1
118	EEF2	eukaryotic translation elongation factor 2
119	EHMT2	euchromatic histone lysine methyltransferase 2
120	EIF3F	eukaryotic translation initiation factor 3 subunit F

Table S5. Genes encoding ESR1-interacting proteins located in differential H3K4me3 peaks

121	EIF4A1	eukaryotic translation initiation factor 4A1
122	EIF4G1	eukaryotic translation initiation factor 4 gamma 1
123	ELMSAN1	None
124	EMD	emerin
125	ENO1	enolase 1
126	EP300	E1A binding protein p300
127	EP400	E1A binding protein p400
128	EPPK1	epiplakin 1
129	ESYT1	extended synaptotagmin 1
130	EWSR1	EWS RNA binding protein 1
131	EXOSC10	exosome component 10
132	EXOSC4	exosome component 4
133	EZH2	enhancer of zeste 2 polycomb repressive complex 2 subunit
134	EZR	ezrin
135	FAF2	Fas associated factor family member 2
136	FAM120A	family with sequence similarity 120A
137	FAM213A	None
138	FASN	fatty acid synthase
139	FIS1	fission, mitochondrial 1
140	FKBP1A	FKBP prolyl isomerase 1A
141	FKBP5	FKBP prolyl isomerase 5
142	FLII	FLII actin remodeling protein
143	FLNA	filamin A
144	FMR1	FMRP translational regulator 1
145	FOS	Fos proto-oncogene, AP-1 transcription factor subunit
146	FOXL2	forkhead box L2
147	FOXO1	forkhead box O1
148	FOXO3	forkhead box O3
149	FTSJ3	FtsJ RNA 2'-O-methyltransferase 3
150	FUS	FUS RNA binding protein
151	G3BP2	G3BP stress granule assembly factor 2
152	GADD45A	growth arrest and DNA damage inducible alpha
153	GADD45B	growth arrest and DNA damage inducible beta
154	GAK	cyclin G associated kinase
155	GAPDH	glyceraldehyde-3-phosphate dehydrogenase
156	GARS	None
157	GLYR1	glyoxylate reductase 1 homolog
158	GNB1	G protein subunit beta 1
159	GNB2	G protein subunit beta 2
160	GNL3	G protein nucleolar 3
161	GOLGA2	golgin A2
162	GRB2	growth factor receptor bound protein 2
163	GSK3B	glycogen synthase kinase 3 beta
164	GTF3C4	general transcription factor IIIC subunit 4
165	H2AFX	None
166	H3F3A	None
167	HAX1	HCLS1 associated protein X-1
168	HDAC1	histone deacetylase 1
169	HDAC2	histone deacetylase 2
170	HDAC3	histone deacetylase 3
171	HDAC4	histone deacetylase 4
172	HDAC5	histone deacetylase 5
173	HEBP2	heme binding protein 2
174	HIF1A	hypoxia inducible factor 1 subunit alpha
175	HIST4H4	None
176	HM13	histocompatibility minor 13
177	HMGB2	high mobility group box 2
178	HNRNPA0	heterogeneous nuclear ribonucleoprotein A0
179	HNRNPA2B1	heterogeneous nuclear ribonucleoprotein A2/B1
180	HNRNPA3	heterogeneous nuclear ribonucleoprotein A3
181	HNRNPAB	heterogeneous nuclear ribonucleoprotein A/B
182	HNRNPD	heterogeneous nuclear ribonucleoprotein D
183	HNRNPF	heterogeneous nuclear ribonucleoprotein F
184	HNRNPK	heterogeneous nuclear ribonucleoprotein K
185	HNRNPL	heterogeneous nuclear ribonucleoprotein L
186	HNRNPM	heterogeneous nuclear ribonucleoprotein M
187	HNRNPR	heterogeneous nuclear ribonucleoprotein R
188	HNRNPU	heterogeneous nuclear ribonucleoprotein U
189	HNRNPUL1	heterogeneous nuclear ribonucleoprotein U like 1
190	HPCAL1	hippocalcin like 1
191	HSP90AA1	heat shock protein 90 alpha family class A member 1
192	HSP90AB1	heat shock protein 90 alpha family class B member 1
193	HSP90B1	heat shock protein 90 beta family member 1
194	HSPA1B	heat shock protein family A (Hsp70) member 1B
195	HSPA1L	heat shock protein family A (Hsp70) member 1 like
196	HSPA4	heat shock protein family A (Hsp70) member 4
197	HSPA5	heat shock protein family A (Hsp70) member 5
198	HSPA8	heat shock protein family A (Hsp70) member 8
199	HSPA9	heat shock protein family A (Hsp70) member 9
200	HSPD1	heat shock protein family D (Hsp60) member 1
201	HSPE1	heat shock protein family E (Hsp10) member 1
202	HSPH1	heat shock protein family H (Hsp110) member 1
203	HUWE1	HECT, UBA and WWE domain containing E3 ubiquitin protein ligase 1
204	HYOU1	hypoxia up-regulated 1
205	IDH2	isocitrate dehydrogenase (NADP(+)) 2
206	ILF3	interleukin enhancer binding factor 3
207	IMMT	inner membrane mitochondrial protein
208	ING1	inhibitor of growth family member 1
209	INTS1	integrator complex subunit 1
210	IPO7	importin 7
211	IQGAP1	IQ motif containing GTPase activating protein 1
212	IRS2	insulin receptor substrate 2
213	JUNB	JunB proto-oncogene, AP-1 transcription factor subunit
214	JUND	JunD proto-oncogene, AP-1 transcription factor subunit
215	KAT2A	lysine acetyltransferase 2A
216	KAT5	lysine acetyltransferase 5
217	KAT6A	lysine acetyltransferase 6A
218	KDM1A	lysine demethylase 1A
219	KDM4A	lysine demethylase 4A
220	KDM4C	lysine demethylase 4C
221	KHDRBS1	KH RNA binding domain containing, signal transduction associated 1
222	KPNB1	karyopherin subunit beta 1
223	LACTB	lactamase beta
224	LARP1	La ribonucleoprotein 1, translational regulator
225	LCK	LCK proto-oncogene, Src family tyrosine kinase
226	LCOR	ligand dependent nuclear receptor corepressor
227	LDB1	LIM domain binding 1
228	LGALS7B	galectin 7B
229	LGALS7B	galectin 7
230	LMNB1	lamin B1
231	LUC7L3	LUC7 like 3 pre-mRNA splicing factor
232	LYAR	Ly1 antibody reactive
233	MAP3K1	mitogen-activated protein kinase kinase kinase 1
234	MAPK14	mitogen-activated protein kinase 14
235	MAPK3	mitogen-activated protein kinase 3
236	MARK2	microtubule affinity regulating kinase 2
237	MBD2	methyl-CpG binding domain protein 2
238	MCM5	minichromosome maintenance complex component 5
239	MDH2	malate dehydrogenase 2
240	MDM2	MDM2 proto-oncogene

Table S5. Genes encoding ESR1-interacting proteins located in differential H3K4me3 peaks

241	MED12	mediator complex subunit 12
242	MED14	mediator complex subunit 14
243	MED16	mediator complex subunit 16
244	MED17	mediator complex subunit 17
245	MED20	mediator complex subunit 20
246	MED25	mediator complex subunit 25
247	MEN1	menin 1
248	MGMT	O-6-methylguanine-DNA methyltransferase
249	MKI67	marker of proliferation Ki-67
250	MKNK2	MAPK interacting serine/threonine kinase 2
251	MMS19	MMS19 homolog, cytosolic iron-sulfur assembly component
252	MOGS	mannosyl-oligosaccharide glucosidase
253	MPG	N-methylpurine DNA glycosylase
254	MRPS2	mitochondrial ribosomal protein S2
255	MRPS27	mitochondrial ribosomal protein S27
256	MRPS6	mitochondrial ribosomal protein S6
257	MSH2	mutS homolog 2
258	MSH6	mutS homolog 6
259	MTA2	metastasis associated 1 family member 2
260	MTA3	metastasis associated 1 family member 3
261	MTCH2	mitochondrial carrier 2
262	MTDH	metadherin
263	MVP	major vault protein
264	MYH9	myosin heavy chain 9
265	MYL6	myosin light chain 6
266	NCL	nucleolin
267	NCOA2	nuclear receptor coactivator 2
268	NCOA3	nuclear receptor coactivator 3
269	NCOA4	nuclear receptor coactivator 4
270	NCOA5	nuclear receptor coactivator 5
271	NCOA6	nuclear receptor coactivator 6
272	NCOA7	nuclear receptor coactivator 7
273	NME2	NME/NM23 nucleoside diphosphate kinase 2
274	NOP2	NOP2 nucleolar protein
275	NOP56	NOP56 ribonucleoprotein
276	NOP58	NOP58 ribonucleoprotein
277	NPEPPS	aminopeptidase puromycin sensitive
278	NR2C2	nuclear receptor subfamily 2 group C member 2
279	NSD1	nuclear receptor binding SET domain protein 1
280	NSDHL	NAD(P) dependent steroid dehydrogenase-like
281	NUP153	nucleoporin 153
282	OTUB1	OTU deubiquitinase, ubiquitin aldehyde binding 1
283	OXSR1	oxidative stress responsive kinase 1
284	P4HB	prolyl 4-hydroxylase subunit beta
285	PABPC1	poly(A) binding protein cytoplasmic 1
286	PAK1	p21 (RAC1) activated kinase 1
287	PCBP1	poly(rC) binding protein 1
288	PCK2	phosphoenolpyruvate carboxykinase 2, mitochondrial
289	PDIA3	protein disulfide isomerase family A member 3
290	PDIA4	protein disulfide isomerase family A member 4
291	PELP1	proline, glutamate and leucine rich protein 1
292	PFKL	phosphofructokinase, liver type
293	PFKP	phosphofructokinase, platelet
294	PFN1	profilin 1
295	PGM2	phosphoglucomutase 2
296	PHB2	prohibitin 2
297	PHGDH	phosphoglycerate dehydrogenase
298	PIAS1	protein inhibitor of activated STAT 1
299	PIAS3	protein inhibitor of activated STAT 3
300	PIK3CA	phosphatidylinositol-4,5-bisphosphate 3-kinase catalytic subunit alpha
301	PIK3R1	phosphoinositide-3-kinase regulatory subunit 1
302	PIK3R2	phosphoinositide-3-kinase regulatory subunit 2
303	PLEC	plectin
304	PNRC2	proline rich nuclear receptor coactivator 2
305	POLR1B	RNA polymerase I subunit B
306	POLR2D	RNA polymerase II subunit D
307	POR	cytochrome p450 oxidoreductase
308	POU2F1	POU class 2 homeobox 1
309	POU2F2	POU class 2 homeobox 2
310	PPARGC1B	PPARG coactivator 1 beta
311	PPP1CA	protein phosphatase 1 catalytic subunit alpha
312	PPP1CC	protein phosphatase 1 catalytic subunit gamma
313	PPP5C	protein phosphatase 5 catalytic subunit
314	PRDM2	PR/SET domain 2
315	PRDX2	peroxiredoxin 2
316	PRDX3	peroxiredoxin 3
317	PRDX6	peroxiredoxin 6
318	PRKAR2A	protein kinase cAMP-dependent type II regulatory subunit alpha
319	PRKCA	protein kinase C alpha
320	PRKCZ	protein kinase C zeta
321	PRKDC	protein kinase, DNA-activated, catalytic subunit
322	PRMT1	protein arginine methyltransferase 1
323	PRMT2	protein arginine methyltransferase 2
324	PRPF4B	pre-mRNA processing factor 4B
325	PSIP1	PC4 and SFRS1 interacting protein 1
326	PSMA5	proteasome 20S subunit alpha 5
327	PSMB9	proteasome 20S subunit beta 9
328	PSMC5	proteasome 26S subunit, ATPase 5
329	PSMD1	proteasome 26S subunit, non-ATPase 1
330	PSMD6	proteasome 26S subunit, non-ATPase 6
331	PTGES3	prostaglandin E synthase 3
332	PTMA	prothymosin alpha
333	PTPN1	protein tyrosine phosphatase non-receptor type 1
334	PTPN6	protein tyrosine phosphatase non-receptor type 6
335	PUS1	pseudouridine synthase 1
336	PYGB	glycogen phosphorylase B
337	RAB10	RAB10, member RAS oncogene family
338	RAB1A	RAB1A, member RAS oncogene family
339	RAB1B	RAB1B, member RAS oncogene family
340	RAB7A	RAB7A, member RAS oncogene family
341	RABGEF1	RAB guanine nucleotide exchange factor 1
342	RAP1A	RAP1A, member of RAS oncogene family
343	RAP2C	RAP2C, member of RAS oncogene family
344	RBBP4	RB binding protein 4, chromatin remodeling factor
345	RBBP6	RB binding protein 6, ubiquitin ligase
346	RBCK1	RANBP2-type and C3HC4-type zinc finger containing 1
347	RBM14	RNA binding motif protein 14
348	RBMX	RNA binding motif protein X-linked
349	RCN2	reticulocalbin 2
350	REEP6	receptor accessory protein 6
351	RELA	RELA proto-oncogene, NF-kB subunit
352	REXO4	REX4 homolog, 3'-5' exonuclease
353	RNF14	ring finger protein 14
354	RNF4	ring finger protein 4
355	RPL10	ribosomal protein L10
356	RPL10A	ribosomal protein L10a
357	RPL13	ribosomal protein L13
358	RPL14	ribosomal protein L14
359	RPL15	ribosomal protein L15
360	RPL18A	ribosomal protein L18a

Table S5. Genes encoding ESR1-interacting proteins located in differential H3K4me3 peaks

361	RPL19	ribosomal protein L19
362	RPL22	ribosomal protein L22
363	RPL28	ribosomal protein L28
364	RPL36AL	ribosomal protein L36a like
365	RPL5	ribosomal protein L5
366	RPL7	ribosomal protein L7
367	RPL7A	ribosomal protein L7a
368	RPL8	ribosomal protein L8
369	RPLP0	ribosomal protein lateral stalk subunit P0
370	RPN1	ribophorin I
371	RPN2	ribophorin II
372	RPS11	ribosomal protein S11
373	RPS14	ribosomal protein S14
374	RPS15A	ribosomal protein S15a
375	RPS19	ribosomal protein S19
376	RPS2	ribosomal protein S2
377	RPS20	ribosomal protein S20
378	RPS23	ribosomal protein S23
379	RPS24	ribosomal protein S24
380	RPS25	ribosomal protein S25
381	RPS3A	ribosomal protein S3A
382	RPS6KA1	ribosomal protein S6 kinase A1
383	RPS6KA3	ribosomal protein S6 kinase A3
384	RPS6KB1	ribosomal protein S6 kinase B1
385	RPS7	ribosomal protein S7
386	RPS8	ribosomal protein S8
387	RRP1B	ribosomal RNA processing 1B
388	RRS1	ribosome biogenesis regulator 1 homolog
389	RSL1D1	ribosomal L1 domain containing 1
390	SAFB	scaffold attachment factor B
391	SAFB2	scaffold attachment factor B2
392	SCYL2	SCY1 like pseudokinase 2
393	SEC11A	SEC11 homolog A, signal peptidase complex subunit
394	SEC22B	SEC22 homolog B, vesicle trafficking protein
395	SEC23A	SEC23 homolog A, COPII coat complex component
396	SEC61B	SEC61 translocon subunit beta
397	SENP5	SUMO specific peptidase 5
398	SETD7	SET domain containing 7, histone lysine methyltransferase
399	SF1	splicing factor 1
400	SIN3A	SIN3 transcription regulator family member A
401	SIN3B	SIN3 transcription regulator family member B
402	SIRT1	sirtuin 1
403	SKI	SKI proto-oncogene
404	SKIL	SKI like proto-oncogene
405	SLC25A5	solute carrier family 25 member 5
406	SLC30A9	solute carrier family 30 member 9
407	SLC3A2	solute carrier family 3 member 2
408	SLC9A3R1	SLC9A3 regulator 1
409	SMAD2	SMAD family member 2
410	SMARCA2	SWI/SNF related, matrix associated, actin dependent regulator of chromatin, subfamily a, member 2
411	SMARCB1	SWI/SNF related, matrix associated, actin dependent regulator of chromatin, subfamily b, member 1
412	SMARCC2	SWI/SNF related, matrix associated, actin dependent regulator of chromatin subfamily c member 2
413	SMARCD1	SWI/SNF related, matrix associated, actin dependent regulator of chromatin, subfamily d, member 1
414	SMARCD3	SWI/SNF related, matrix associated, actin dependent regulator of chromatin, subfamily d, member 3
415	SMURF1	SMAD specific E3 ubiquitin protein ligase 1
416	SNRPD1	small nuclear ribonucleoprotein D1 polypeptide
417	SOS1	SOS Ras/Rac guanine nucleotide exchange factor 1
418	SOS2	SOS Ras/Rho guanine nucleotide exchange factor 2
419	SP1	Sp1 transcription factor
420	SP2	Sp2 transcription factor
421	SP3	Sp3 transcription factor
422	SPOP	speckle type BTB/POZ protein
423	SPTAN1	spectrin alpha, non-erythrocytic 1
424	SREBF1	sterol regulatory element binding transcription factor 1
425	SRPK1	SRSF protein kinase 1
426	SRRM1	serine and arginine repetitive matrix 1
427	SRRM2	serine/arginine repetitive matrix 2
428	SRSF1	serine and arginine rich splicing factor 1
429	SRSF2	serine and arginine rich splicing factor 2
430	SRSF5	serine and arginine rich splicing factor 5
431	SRSF6	serine and arginine rich splicing factor 6
432	SRSF7	serine and arginine rich splicing factor 7
433	STAT3	signal transducer and activator of transcription 3
434	STAT5A	signal transducer and activator of transcription 5A
435	STAU1	staufen double-stranded RNA binding protein 1
436	STRN	striatin
437	STUB1	STIP1 homology and U-box containing protein 1
438	SUMO1	small ubiquitin like modifier 1
439	SYNCRIP	synaptotagmin binding cytoplasmic RNA interacting protein
440	TAB2	TGF-beta activated kinase 1 (MAP3K7) binding protein 2
441	TADA3	transcriptional adaptor 3
442	TAF1B	TATA-box binding protein associated factor, RNA polymerase I subunit B
443	TAGLN2	transgelin 2
444	TAP1	transporter 1, ATP binding cassette subfamily B member
445	TBL2	transducin beta like 2
446	TBP	TATA-box binding protein
447	TCF20	transcription factor 20
448	TCF7L1	transcription factor 7 like 1
449	THEM6	thioesterase superfamily member 6
450	THRAP3	thyroid hormone receptor associated protein 3
451	TKT	transketolase
452	TMED10	transmembrane p24 trafficking protein 10
453	TMED2	transmembrane p24 trafficking protein 2
454	TMOD3	tropomodulin 3
455	TMX1	thioredoxin related transmembrane protein 1
456	TNFRSF14	TNF receptor superfamily member 14
457	TNPO2	transportin 2
458	TOP1	DNA topoisomerase I
459	TOP2B	DNA topoisomerase II beta
460	TOR1AIP1	torsin 1A interacting protein 1
461	TPM1	tropomyosin 1
462	TRAF6	TNF receptor associated factor 6
463	TRAM1	translocation associated membrane protein 1
464	TRAP1	TNF receptor associated protein 1
465	TRIM24	tripartite motif containing 24
466	TRIM25	tripartite motif containing 25
467	TRIM59	tripartite motif containing 59
468	TRIP12	thyroid hormone receptor interactor 12
469	TRIP4	thyroid hormone receptor interactor 4
470	TRRAP	transformation/transcription domain associated protein
471	TSC2	TSC complex subunit 2
472	TUBA1A	tubulin alpha 1a
473	TUBA1C	tubulin alpha 1c
474	TUBB	tubulin beta class I
475	TUBB2A	tubulin beta 2A class IIa
476	TUBB4B	tubulin beta 4B class IVb
477	U2AF2	U2 small nuclear RNA auxiliary factor 2
478	UBA3	ubiquitin like modifier activating enzyme 3
479	UBAP2L	ubiquitin associated protein 2 like
480	UBC	ubiquitin C

Table S5. Genes encoding ESR1-interacting proteins located in differential H3K4me3 peaks

481	UBE2I	ubiquitin conjugating enzyme E2 I
482	UBE3A	ubiquitin protein ligase E3A
483	UBTF	upstream binding transcription factor
484	UGDH	UDP-glucose 6-dehydrogenase
485	UIMC1	ubiquitin interaction motif containing 1
486	UNC45A	unc-45 myosin chaperone A
487	UPF1	UPF1 RNA helicase and ATPase
488	USF1	upstream transcription factor 1
489	UTP14A	UTP14A small subunit processome component
490	UTP3	UTP3 small subunit processome component
491	VAPA	VAMP associated protein A
492	VAPB	VAMP associated protein B and C
493	VCL	vinculin
494	VCP	valosin containing protein
495	VDAC1	voltage dependent anion channel 1
496	VDAC3	voltage dependent anion channel 3
497	WDR1	WD repeat domain 1
498	WDR18	WD repeat domain 18
499	WDR5	WD repeat domain 5
500	WIPI1	WD repeat domain, phosphoinositide interacting 1
501	XBP1	X-box binding protein 1
502	XPO1	exportin 1
503	XPO5	exportin 5
504	XRCC6	X-ray repair cross complementing 6
505	YBX1	Y-box binding protein 1
506	YTHDF2	YTH N6-methyladenosine RNA binding protein 2
507	YWHAB	tyrosine 3-monooxygenase/tryptophan 5-monooxygenase activation protein beta
508	YWHAE	tyrosine 3-monooxygenase/tryptophan 5-monooxygenase activation protein epsilon
509	YWHAQ	tyrosine 3-monooxygenase/tryptophan 5-monooxygenase activation protein theta
510	YWHAZ	tyrosine 3-monooxygenase/tryptophan 5-monooxygenase activation protein zeta
511	ZBTB17	zinc finger and BTB domain containing 17
512	ZC3HAV1	zinc finger CCCH-type containing, antiviral 1
513	ZNF398	zinc finger protein 398
514	ZNFX1	zinc finger NFX1-type containing 1
515	ZNHIT3	zinc finger HIT-type containing 3

Table S6. Genes encoding DNA repair proteins located in differential H3K4me3 peaks

	gene	Description
1	ABL1	ABL proto-oncogene 1, non-receptor tyrosine kinase
2	ATM	ATM serine/threonine kinase
3	ATR	ATR serine/threonine kinase
4	ATRIP	ATR interacting protein
5	BACH1	BTB domain and CNC homolog 1
6	BARD1	BRCA1 associated RING domain 1
7	BLM	BLM RecQ like helicase
8	BRCC3	BRCA1/BRCA2-containing complex subunit 3
9	CDK9	cyclin dependent kinase 9
10	CHEK2	checkpoint kinase 2
11	FANCA	FA complementation group A
12	MDC1	mediator of DNA damage checkpoint 1
13	MLH1	mutL homolog 1
14	MORF4L1	mortality factor 4 like 1
15	MSH2	mutS homolog 2
16	MSH3	mutS homolog 3
17	MSH6	mutS homolog 6
18	NBN	nibrin
19	PIAS4	protein inhibitor of activated STAT 4
20	POLR2I	RNA polymerase II subunit I
21	PRKDC	protein kinase, DNA-activated, catalytic subunit
22	RAD23B	RAD23 homolog B, nucleotide excision repair protein
23	RAD51	RAD51 recombinase
24	RBX1	ring-box 1
25	RFC2	replication factor C subunit 2
26	RPA1	replication protein A1
27	SIRT1	sirtuin 1
28	SMC1A	structural maintenance of chromosomes 1A
29	SUMO1	small ubiquitin like modifier 1
30	TP53BP1	tumor protein p53 binding protein 1
31	TRRAP	transformation/transcription domain associated protein
32	UBE2D3	ubiquitin conjugating enzyme E2 D3
33	UBE2N	ubiquitin conjugating enzyme E2 N
34	UBE2W	ubiquitin conjugating enzyme E2 W
35	UIMC1	ubiquitin interaction motif containing 1
36	VCP	valosin containing protein
37	WDR48	WD repeat domain 48
38	XRCC6	X-ray repair cross complementing 6
39	YY1	YY1 transcription factor

Table S7. Primers used for repeat, Chip-qPCR and Medip-qPCR analysis

	CNV		
LINE L1 ORF2-1	TGCGGAGAAATAGGAACACTTTT	TGAGGAATCGCCACACTGACT	[1]
LINE L1 ORF2-2	CAAACACCGCATATTCTCACTCA	CTTCCTGTGTCCATGTGATCTCA	[1]
SAT2	CATCGAATGGAAATGAAAGGAGTC	ACCATTGGATGATTGCAGTCAA	[2]
SATA	CTGCACTACCTGAAGAGGAC	GATGGTTCAACACTCTTACA	[2]
ALU	CCAGAAAAAATTACCAATTAGTTC	GGGCCTATTGACTATGCTTAC	[3]
Telomere length	CGGTTTGTTTGGGTTTGGGTTTGGGTTTGGGTTTGGGTT	GGCTTCCCTTACCCTTACCCTTACCCTTACCCTTACCCT	
RPLP0	CAGCAAGTGGGAAGGTGTAATCC	CCCATTCTATCATCAACGGGTACAA	
	DNA methylation		
ARID2	GGCCGAAATACACGCTGTCA	CGATGGTGAGGGGTCTGGG	*
ATM	CTGCGTGGCTAACGGAGAAA	TACCACTGCACTCGGAAGGT	*
EGFR	GCCCAGACCGGACGACA	ATACTGGACGGAGTCAGGGG	*
ESR2	CAGTGTGGACGCCTACGAG	GATTTTGTCACCCGCACGTC	*
ESRRA	GCAACTTCCCAAAGGTGTGC	AGCCTGCAGGTCCACTCTTA	*
H19	CTCCTTGCTGCGCAATGTCC	GGAAGACAGGCAGTGCTCG	*
H2AFX	ACGGTGGCGCTGGTCTTC	CCATCCGCAACGACGAGG	*
IGF2	TCGTCTGATTGTCCAGGGAGGA	CTGACCTCATTTCCCGATACCTTT	*
KAT5	GCGGAACCAGGACAACGAA	GGGATTTCAGTTGACTCCCGC	*
KCNQ1	AACCACGATGACTGACGCAC	GCGATTCTGCCGGGGTG	*
LINE1	TCTGAGCCAGGTGTGGGATA	TCACTAGGGAGTGCCAGACA	[4]
OGG1	CCTACACCTCAGGAAAGCCG	GTGCTGTTTAACAACCTTCCCTG	*
PGR	TTATCTTTAGAGCGGGCGGC	GGAGTTCACCCTGTGCCTC	*
RPLP0	CAGCAAGTGGGAAGGTGTAATCC	CCCATTCTATCATCAACGGGTACAA	*
SAT2	CATCGAATGGAAATGAAAGGAGTC	ACCATTGGATGATTGCAGTCAA	*
SINEB1	TGGTGGTGCATGCCTTTAAT	CCTGGTGTCCTGGAACTCACT	[3]
SMG7	GTGGACTTCACCACCGGGGA	AACCGATGTGTGCGGACAGG	*
SUV39H1	AAAGGTGAAGCAGTGGAGGC	CCTCATCCTTTCCGCGCC	*
TERF2IP	GACACCGGCTCGGAAGCAAA	ATGGCTACGTCGTCCGCATC	*
TERT	CAGGCGCCGCACGAA	CACCCCCGCGATGCC	*
UPF1	TACGCCAGCTGGGTTTGCTC	GACCAGGGCAGGTCACCCTA	*
UPF2	CTGAGGAGAACGACGCCGGA	GCTCCAACGATCGGGAGGAA	*
VEGFA	CAGCTACCACCTCCTCCCC	CAGCCCAGAAGTTGGACGAA	*
XRCC5	GCCTGAGGACCGGCAACAT	TTCCTGCTTCCGAACCACCC	*
ZBTB48	GCCGCTCGGGACCTGTG	ATGAGAGCGAACGGACTGGC	*
	ChIP		
STAT2	TCAGGGCCCGTACCTGATTA	GGGGCGCGAGGTTCTAGT	[4]
5'UTR LINE	GCCAAGATGGCCGAATAGG	AAATCACCCGTCTTCTGCGT	[6]
BRCA1	TTCCCTCGCGACCTACAAAC	CGTGGGAGAGTGGATTTCCG	
ESR1	ACTCAACAGCGTGTCTCCGA	GGGCTCGTTCTCCAGGTAGT	
KMT2E	GGGGTCGAGCACATAACGG	ACACAAGTACCCCAACCCCG	[4]
RARA	CGGGTAAAGTTTCAGCCTCCG	GCGTCTGCCCTAACCCG	

* used for DNA methylation and ChIP-qPCr analysis

1. Coufal NG, Garcia-Perez JL, Peng GE, Yeo GW, Mu Y, Lovci MT, et al. L1 retrotransposition in human neural progenitor cells. Nature. 2009;460:1127–31.

2. Alexiadis V, Ballestas ME, Sanchez C, Winokur S, Vedanarayanan V, Warren M, et al. RNAPol-ChIP analysis of transcription from FSHD-linked tandem repeats and satellite DNA. Biochim Biophys Acta. 2007;1769:29–40.

3. Varshney D, Vavrova-Anderson J, Oler AJ, Cowling VH, Cairns BR, White RJ. SINE transcription by RNA polymerase III is suppressed by histone methylation but not by DNA methylation. Nat Commun. 2015;6:6569.

4. Legoff L, D'Cruz SC, Bouchekhchoukha K, Monfort C, Jaulin C, Multigner L, et al. In utero exposure to chlordecone affects histone modifications and activates LINE-1 in cord blood. Life Sci Alliance. 2021;4:e202000944.

5. Dessain SK, Yu H, Reddel RR, Beijersbergen RL, Weinberg RA. Methylation of the human telomerase gene CpG island. Cancer Res. 2000;60:537–41.

6. De Cecco M, Ito T, Petrashen AP, Elias AE, Skvir NJ, Criscione SW, et al. L1 drives IFN in senescent cells and promotes age-associated inflammation. Nature. 2019;566:73–8.

Table S8. ChIP-seq reads

Group	Sample name	BPA (microg/gram Creat)	number of reads
low	FMSV217	2,55	4,64E+07
low	FMSV218	1,12	6,22E+07
low	FMSV219	2.13	4,94E+07
low	FMSV220	2,43	4,46E+07
low	FMSV221	0,66	4,24E+07
low	FMSV222	1,10	6,40E+07
low	FMSV223	0,73	4,97E+07
low	FMSV224	2,31	4,62E+07
low	FMSV225	2,35	5,27E+07
low	FMSV226	1,28	6,70E+07
high	FMSV167	19,57	4,37E+07
high	FMSV170	17,11	5,20E+07
high	FMSV173	10,55	6,20E+07
high	FMSV175	39,34	4,89E+07
high	FMSV177	42,64	5,47E+07
high	FMSV233	10,07	5,49E+07
high	FMSV234	12.24	6,41E+07
input	FMSV 178	input	6,38E+07

Table S9. Primers used for RT-qPCR in this work

	forward	reverse
ARID2	GGAGCAGATCCAAGCACTGT	TGGCATAGGAGTGTTCTGCG
ATM	CCGAGTGCAGTGACAGTGAT	CATGTTCTAGTTGACGGCAGCA
BRCA1	TCAGGAGGCCTTCACCCTC	TTGATCAACTCCAGACAGATGGG
CDCA5	ATAAGAGCCTGAAGGAGGGAGCC	TCTTGGCTGTTGTCTCCCTGAGC
DNMT1	ACAGAAAAGGAATGTGTGAAGGAGA	CCAGGTAGCCCTCCTCGGAT
EGFR	ACACCTTCCTCCCAGTGCCT	CCCCCTCTGGAGATGCTGGA
ESR1	CCCAGGGAAGCTACTGTTTGC	TGATGTAGCCAGCAGCATGTC
ESR2	CAAACCAAAGCATCGGTCACG	AGGCCATGATCCTGCTCAATTC
ESRRA	AGAGGACCATCCAGGGGAGC	CTCCTTGAGCATGCCCACCC
EZH2	GACACCCGGTGGGACTCAGA	AGTCTGGCCCATGATTATTCTTCGT
FOS	CACTCCAAGCGGAGACAGAC	GTGAGCTGCCAGGATGAA
HDAC1	CCTGGGTCGGACGCTGAG	AATTTCCAACATCCCCGTCGT
HDAC2	GTCTGCTACTACTACGACGGTGATA	TTAACAGCAAGTTATGGGTCATGCG
HDAC5	ACGAGTCGGCAGATGGGATG	ACCGGCTTCACCTCCACTGT
HDAC6	AGTCGCGGGGAAAAGGTCG	GGTGGAATCCTGGCCGGTTG
IGF2	CTGGCAGAGGAGTGTCCGGC	GGGATTCCCATTGGTGTCTGGAAG
KAT5	GAGGTGGGTAGAGCCCGAGG	GATCTCGGCCAGGGGCCA
KDM1A	AGCGAATCCCCCAAGTGATGT	AGAGAGTTGAGAGAGGTGTGGCA
KMT2E	GGTCGAGCACTGTGGCTG	CTCATGACGTTCGCCTCTGG
NAPBL	GGCTGGCTTGGTGGGGTG	CGGCCTGTCTCCAGCACAAT
NUDT1	CCGACGACAGCTACTGGTTT	CGCGGAGTGTGTAGTCCAG
OGG1	TTTCTGCGCTTTGCTGGTGG	CTGTGGGGACCTTATGCTGG
PGR	GGCATGGTCCTTGGAGGTCG	ATTTGGAACGCCCACTGGCT
POT1	GGCGGAGAACAAGCGACTAT	ATCTGACAACTTGCTGCCGT
RAD18	ATCCTCTTCTCTGTCAGGTTCCA	CTATCTTCTGTATGCATGGGACAGG
RAD21	GCTTCATGGTCTTCAGCGTGC	AGAACTTTGCGGCAGCTTGT
RPL37A	GAGACGAGCTGTGGGGATCT	CTGATGGCGGACTTTACCGT
SETDB1	TGGAAGACGGGAGAGGACAA	GTTCCTCAACCACTGCCTGT
SMG1	TGACTGGCAACCCAGAACTGA	GCCGAGACACCACAGCTGAA
SMG7	GAGCCTGAGAGACCCACGGA	GGACCCAGCTTAGAATCGGAGG
STAT2	CGTGTACGGTTGCACAGAGT	GGCACCTTTCTACTGCGCTT
TEN1	GACGCCTAGAACAGGAGCCC	ATGTTCTCAGCGTGCTCCCA
TERF2IP	GCGGATAGCGGGGAACCAC	CCTCAAACTCCCGGGTGGC
TERT	TGGGGTTCTTCCAAACTTGCT	GACGGTGTGCACCAACATCT
TP53BP1	TCGTGCCTCCATGGGAGTTCT	AACTCTCCTGCCCCTACAGGTT
UPF1	AGGTCCCTGATAATTATGGCGATG	TGCGCTCTGCATCCTGTCAA
UPF2	CAACAGGCTCGAGATTGTCCT	TTGGCCTCTCCTTGCTGCTC
VEGFA	GGTGCCCGCTGCTGTCTAAT	CCTTGCAACGCGAGTCTGTG
XRCC5	ACCATGTTTGTACAGCGACAGG	CCCCACCAGAAAGGGGATTG
XRCC6	CGCTTCGCTCGTGAGCAGT	AGTCTCCACTTGCTTCAAGGTTCT
XRCC6	CGCTTCGCTCGTGAGCAGT	AGTCTCCACTTGCTTCAAGGTTCT
ZBTB48	CTGTCGGCGGGGAGCTTT	CCTTCTCCCGCTGCTTGTTGA
